# The Efficacy of Methane Leak Detection and Repair (LDAR) Programs in Practice

**DOI:** 10.1021/acsestair.5c00195

**Published:** 2025-10-28

**Authors:** Shona E. Wilde, David R. Tyner, Matthew R. Johnson

**Affiliations:** Energy and Emissions Research Laboratory, Department of Mechanical and Aerospace Engineering, 6339 Carleton University, Ottawa, Ontario K1S 5B6, Canada

**Keywords:** methane, LDAR, OGI, aerial, oil and gas, fugitive emissions, compliance, reconciliation

## Abstract

Periodic leak detection and repair (LDAR) surveys are a key part of most modern oil and gas sector methane regulations, however their effectiveness in real-world practice has been difficult to assess. This study analyzes three years of reported data from regulated LDAR surveys in British Columbia, Canada, which suggest that 3×/year optical gas imaging (OGI)-based LDAR surveys reduce detected emissions by half at fully compliant sites. However, independent source-resolved aerial surveys at an identical subset of sites find 12 times more methane emissions overall, and four times more emissions after conservatively excluding potential combustion-related and intentional vent sources not targeted by OGI LDAR surveys. This demonstrates that regulated OGI-based LDAR surveys only capture a small portion of total emissions in practice, raising concerns about overestimated mitigation impacts and potentially misguided expectations when assessing alternative technologies. Further analysis reveals the two methods find complementary subsets of sources, with aerial detections comprising a range of larger combustion, vent, and fugitive sources and LDAR detections dominated by numerous smaller leaks from connectors and valves. This underscores the importance of integrating complementary measurement approaches to capture the full distribution of emissions and the necessity of independent verification frameworks such as OGMP 2.0.

## Graphical abstract

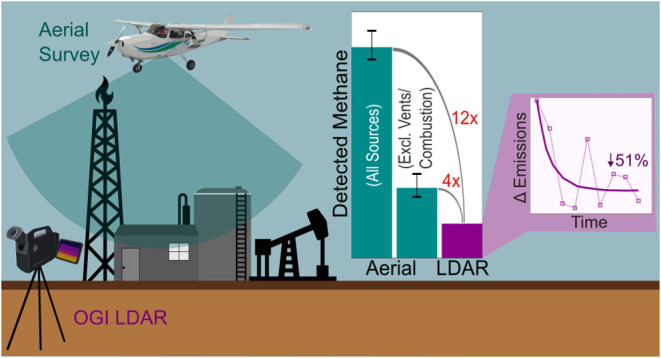

## 1 Introduction

The oil and gas industry is a primary source of anthropogenic methane emissions, with many recent measurement studies suggesting reported emissions in national inventories are underestimated by 50–100%.^1–8^ However, this sector is also understood to have the greatest near-term mitigation potential.^9,10^ Immediate mitigation of oil and gas methane emissions is thus a key focus of international efforts to rapidly reduce atmospheric methane by 2030, which is critical for holding global temperature rise below 1.5–2 °C.^11–13^ The US and Canada have implemented and continue to develop regulations to address methane emissions from the upstream oil and gas sector^14–16^ and similar regulations have been introduced in the European Union.^17^ These policies typically combine limits on intentional venting of gas with prescribed leak detection and repair (LDAR) programs intended to find and fix sources of fugitive emissions, i.e., unintentional or unknown releases of methane to atmosphere.^15,18,19^

In a typical LDAR program, surveys are performed multiple times per year to detect leaks via optical gas imaging (OGI) using hand-held infrared cameras or using a gas concentration meter as outlined in EPA’s Method 21.^20^ Operators are typically required to repair detected leak sources within a designated time frame. Although frequent LDAR programs have been shown to be important for reducing emissions in simulations,^21^ few studies have attempted to assess the mitigation effectiveness of LDAR surveys in practice. This is especially important in the context of proposed and emerging alternative fugitive emissions management programs which are typically evaluated against an assumed baseline performance of traditional OGI or Method 21 LDAR.^22,23^ Critically, uncertainties in the real-world performance of existing LDAR programs hinder development and evaluation of potentially improved methods that substitute or incorporate alternative technologies.^24–27^

Tyner and Johnson^28^ contrasted aerial gas mapping LiDAR measurements with OGI survey data collected one-year prior at the same set of 140 sites. They found surprising differences between the two data sets, with the aerial system finding much higher magnitudes, different types, and a smaller number of sources than OGI. However, while this highlighted the potential limits of both technologies, as the OGI surveys were collected one year prior and not specifically as part of a regulated LDAR campaign, the results do not speak directly to the efficacy of LDAR in reducing fugitive emissions.

Cheadle et al.^29^ analyzed operator-reported data from the first two years of regulated quarterly EPA Method 21 LDAR surveys (a protocol for fugitive source detection but not quantification)^20^ at facilities in California during 2018 and 2019. Results showed decreasing trends in the number of detected leaks and an overall decrease in the number of detected leaks per surveyed component. Using emission factor correlations and time of repair data, they estimated that this led to methane reductions equivalent to 9% and 4% of total inventoried oil and gas production and processing emissions (including unintentional leaks, intentional venting, and methane emitted from combustion processes) in each year. However, without an independent measure of emissions it is difficult to discern if any portion of emissions may have been missed during surveys or what impact the LDAR program had on fugitive sources specifically.

Ravikumar et al.^30^ conducted initial OGI-based LDAR surveys at 36 sites in Alberta, Canada and follow-up surveys at 8 of these sites 6–13 months afterward. While the initial surveys identified many sources, tank venting was dominant, such that fugitive leaks (i.e., the focus of most LDAR programs) were only estimated to comprise <15% of observed methane emissions (noting also that combustion sources such as methane-slip from compressors were not considered). This is consistent with the findings of Tyner and Johnson^28^ as well as recent national-scale source-resolved aerial surveys, where across western Canada venting and combustion sources were found to dominate upstream oil and gas sector methane emissions.^2–4^ During resurveys at the subset of 8 revisited sites, Ravikumar et al.^30^ found that more than 90% of leaks from the initial surveys had been repaired. However, this only resulted in a 22% decrease in total fugitive emissions, with emissions actually increasing for 2 of 8 sites in the sample. This was attributed to the occurrence of new sources (or previously missed sources) from the initial surveys, highlighting the importance of frequent LDAR in managing fugitive emissions. However, notwithstanding the very small sample size, the apparent 22% reduction is just over half the predicted 40% emissions reduction in the seminal simulation study^21^ that underpins prescribed frequencies in many current regulations.^31,32^

One reason for this difference may be poorer than expected performance of OGI cameras in practice. In controlled tests of OGI surveyors,^33^ achieved detection rates were significantly lower and required leak sizes to achieve 90% detection probability were an order of magnitude higher than predicted in prior studies based on camera performance. This was true even though surveyors “tended to be strongly focused on their detection tasks, and typically exhibited a competitive spirit to detect as many leaks as possible, in an environment where they knew there would be leaks”.^33^ Controlled test data also revealed large differences in detection rates based on the level of surveyor experience.

A further determinant of the success of LDAR programs is operator compliance. A review^34^ of industry-submitted LDAR data in the province of British Columbia (BC), Canada for 2020 found very poor compliance rates, with only 28% of facilities and 62% of wells fully meeting the regulatory requirements for number and type of LDAR surveys completed. However, as further discussed below, there are several challenges with the accuracy and currency of public facility and well activity data in BC that likely affected these estimates. The authors^34^ also specifically noted that their results were likely affected by the COVID-19 pandemic as well as the study period being the first year for newly required LDAR regulations. Cheadle et al.^29^ similarly noted poorer compliance at facilities subject to California state-wide LDAR requirements in the first year of regulations, with a 36% increase in the number of participating operators and a 23% increase in the number of facilities submitting reports in the second year of the program.

In the context of limitations and uncertainties in real-world LDAR performance raised in previous studies, the objectives of this work were to (i) analyze three-years of regulated, industry reported LDAR data for oil and gas sites in the province of British Columbia, Canada to quantify compliance rates, detected sources distributions, and trends, (ii) to contrast reported LDAR survey data with independent aerial measurements completed at a matched subset of approximately 500 operating oil and gas sites, and (iii) to analyze observed discrepancies in magnitudes, types, and distributions of sources to understand the effectiveness and limits of both approaches under real-world conditions. This presented analysis represents one of the first large scale tests of regulatory LDAR programs in practice, providing quantitative insight into limits of current LDAR, support for the importance of independent emissions verification under emerging monitoring, reporting, and verification (MRV) programs, and guidance for the design of potential alternative LDAR programs under emerging regulatory scenarios.

## 2 Methods

### 2.1 Data

#### 2.1.1 Leak, Detection and Repair (LDAR)

Regulated LDAR survey data collected during 2020, 2021, and 2022 for facilities and wells in BC were obtained from the British Columbia Energy Regulator (BCER).^35^ As further explained in the Supporting Information (SI), since 2020, facilities and wells in BC have been required to complete 1× or 3× per year surveys at most facilities and wells and submit detailed reports that are aggregated and shared publicly.^35^ Submitted information includes:^36^

- The facility identifier (Kermit ID) or Well Authorization (WA) number;
- Survey metadata including survey date, meteorological conditions, whether the survey was conducted by internal personal or by a third-party service company, survey method (e.g., OGI or Method 21 for comprehensive surveys, or auditory, visual, olfactory (AVO) for screening surveys), and make and model of employed measurement devices.
- Leak information, including whether the leak is situated within a building, the specific process or equipment block where the leak is found (e.g., compressor, tank), type of leaking component (e.g., connector, valve, regulator, pressure relief valve), the leak rate, and method of quantification (e.g., Hi-Flow Sampler, quantification using quantitative OGI (QOGI), or estimation using emission factors).
- Information on leak repair, including the repair date and specifics on how the leak was addressed (e.g., tightened fitting or replaced component).

Importantly, the guideline ensures that even surveys where no measurable leaks are detected are recorded, allowing for comprehensive compliance assessment.

#### 2.1.2 Aerial Surveys

Aerial measurements^2^ using Bridger Photonics’ Gas Mapping LiDAR (GML) were completed during September 11 to October 8, 2021, over 508 distinct sites (i.e., distinct pads or production sites containing one or more facilities and wells as defined for reporting purposes) in BC, Canada. A stratified sampling approach ensured these sites were representative of the variety of upstream oil and gas facilities in the province and included a range of active well sites, single- and multiwell oil and gas facilities, compressor stations, gathering facilities, and gas plants.^2,37^ As described in-depth elsewhere,^28,38,39^ the GML employs a scanning laser and camera mounted on a light aircraft, providing high-resolution (1–2 m), quantitative, geo-located imagery of methane plumes. Source emission rates are estimated using proprietary processing techniques considering plume height above ground, the spatial concentration of CH_4_, and locally estimated wind speed data, typically sourced from the public High-Resolution Rapid Refresh (HRRR) database (NOAA, 2020) or Meteoblue (http://meteoblue.com). For the first generation GML technology (GML 1.0) used in the present work, robust, independently derived probability of detection (POD) and quantification uncertainty models suggest that at the typical altitude of 175 m above ground level and 3-m windspeeds between 1.7 and 8.3 m/s (95% equal tail confidence interval), sources between 0.7–3.5 and 1.5–7.1 kg/h will be detected with 50% and 90% probability, respectively.^38^ All sites with detected sources were reflown at least once on separate days. Emission rates and uncertainties for each detected source were derived from pass-by-pass aerial data via a combined Monte Carlo and Bayesian analysis.^2^ The Monte Carlo analysis perturbs each successfully detected and quantified source in each pass according to an independently derived quantification error model for Bridger’s GML 1.0,^38^ while the potential for missed detections of sources seen in some but not all passes (whether due to source variability, intermittency, or the finite, condition-specific probability of detection^38^) are simultaneously considered within a Bayesian framework.^2^

### 2.2 Determining Compliance

In BC, fugitive emissions surveys are required at facilities and wells active (i.e., pressurized in whole or in part) for more than 30 or 90 days respectively within a calendar year. As detailed in the SI, accurate, monthly lists of active facilities and wells during 2020, 2021, and 2022 were compiled by linking monthly volumetric data reported by industry through the Petrinex system^40^ with the KERMIT facility and well identifiers used when submitting LDAR reports. Since multiple facilities and wells (each identified by a separate KERMIT ID or WA number) are often colocated on a single pad, compliance rates presented in the main text were calculated on a site-by-site basis, with additional scenarios presented in the SI. Each site was defined by a polygon covering the pad’s extent. These polygons were defined as “facilities” if they contained infrastructure with a KERMIT ID for a facility type (e.g., compressor station, battery, gas plant, etc.) or “wells” if they contained only well infrastructure. Consistent with BC regulations,^41^ the assumed requirements for 1× or 3×/year comprehensive or 1×/year screening surveys considered the type(s) of facility­(ies) at each site, the type of production (conventional or unconventional), whether controlled or uncontrolled storage tanks were present, and the number of days the facility or well was pressurized. Full details on the specific requirements for survey types and frequencies are outlined in the Supporting Information (Section S1.2).

## 3 Results and Discussion

### 3.1 Compliance Rates

LDAR compliance rates are a crucial determinant of regulatory effectiveness and must be understood prior to comparing LDAR detections with those of alternative technologies. As detailed in the SI and summarized in Figure 1, compliance rates for active facility and well sites have risen substantially since the introduction of regulations in 2020 but remain less than perfect. As of 2022, 86% of facilities completed all required comprehensive LDAR surveys, and 96% were surveyed at least once. However, only 58% of the of the 53 active compressor stations reporting to the KERMIT system completed all required comprehensive surveys (Figure S2 and Table S8), and 28% submitted no surveys. This is notable given the importance of compressor-related emissions to total upstream methane emissions in BC.^2,28^ Across all active facilities, the same two operators accounted for at least 40% of sites with no submitted surveys in 2020, 2021 and 2022, resulting in ongoing enforcement investigations initiated by BCER.^42^

**Figure 1:**
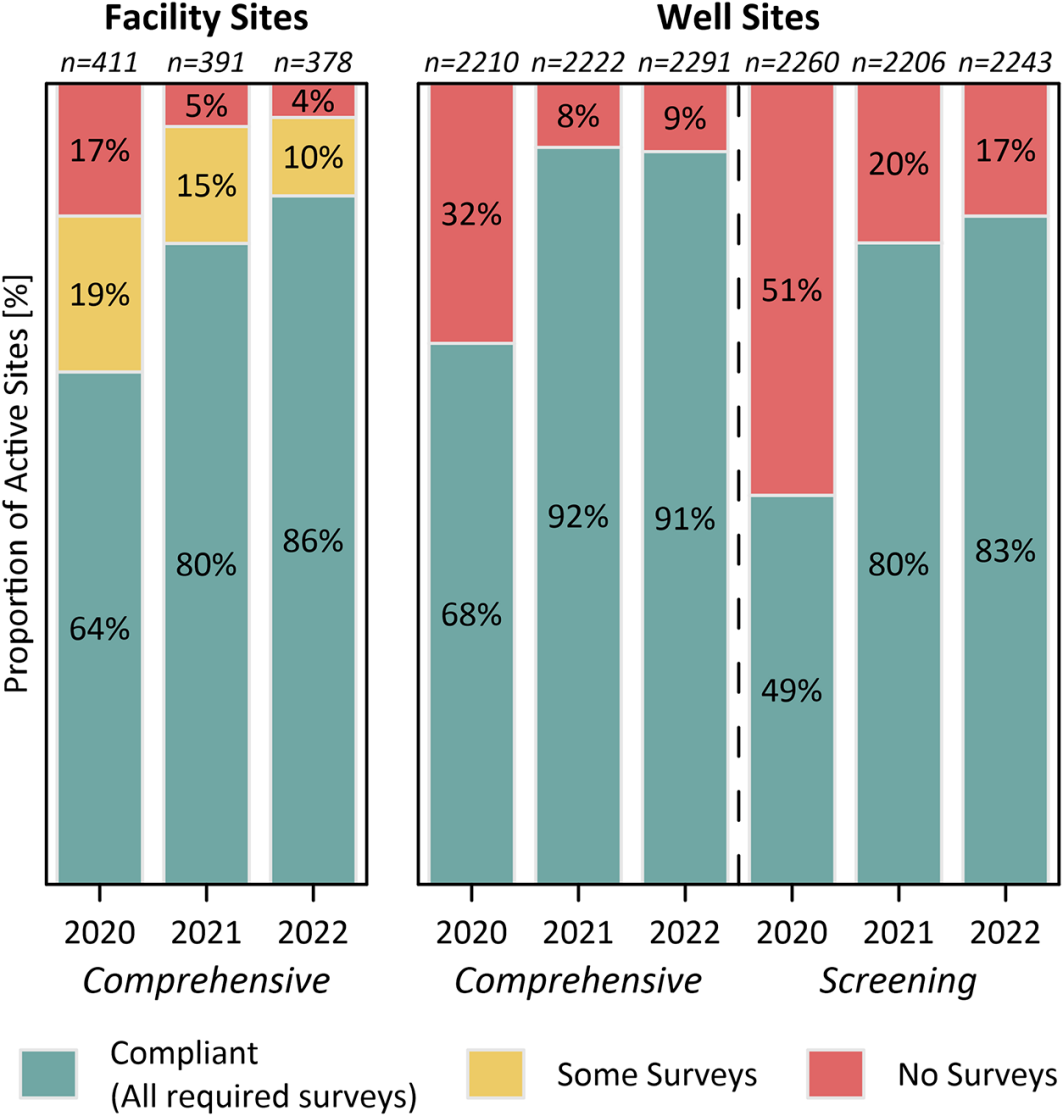
Compliance statistics for active facility and well sites (pads) in BC based on the required survey type (comprehensive or screening), where facilities require 1–3 surveys per year (See SI) and wells require one survey per year. See SI Figure S3 for additional data on the small number of facilities requiring a single annual screening survey.

For well sites requiring comprehensive LDAR surveys, 91% complied with regulations in 2022, whereas 83% of sites requiring screening surveys were compliant (Table S9). In both cases, the majority of noncompliant sites were single-well sites, characterized by a single wellhead (typically linked to a separate central production facility via a pipeline) without any other officially identified on site infrastructure. Specifically, of the 195 well sites missing comprehensive LDAR surveys, 84% (163) were single-well sites. Similarly, of the 371 well sites missing screening surveys, 96% (356) were single-well sites. Overall, on a per-well basis, 92.5% of the 10,179 active wells in 2022 were estimated to be compliant with regulations.

Although the presently derived facility compliance rate of 64% for 2020 (the first year of regulations) is low, it is much higher than a previous analysis that estimated only 28% of facilities (357 of 1276) were fully compliant in 2020.^34^ This difference is almost entirely attributable to improved estimates of active facilities as fully detailed in the SI. In particular, BCER has acknowledged that the status of facilities (i.e., active, inactive, suspended, removed, etc.) in current public facility lists may not be accurate or may be delayed in being updated,^43^ and the analysis summarized in Table S8 ultimately finds a much lower count of active facilities (814 identified facilities located within 411 sites) during 2020, of which 636 were determined to require surveys under current regulations. Estimated compliance for wells in 2020, whose active status is more easily tracked using production data, were more similar—58% on a per well-site basis (combining the percentage of compliant well sites requiring comprehensive and screening surveys from Figure 1) or 71% on a per well basis using data from Table S8 versus the previously estimated 62%.^34^ Most importantly, it is apparent that the low compliance in 2020 (likely due in part to the COVID-19 pandemic and the concurrent economic and logistical challenges faced by operators adapting to the new regulations as previously noted^34^), has since improved.

### 3.2 Multiyear Trends in Detected Leaks and Emissions via Regulated LDAR

Since the start of regulations in 2020 through the end of 2022, most facility sites in BC have completed up to nine consecutive comprehensive LDAR surveys, and well sites have completed three consecutive comprehensive or screening surveys. At facilities subject to 3×/yr comprehensive LDAR, as reported, both the mean number of detected leaks per site (Figure 2a) and the mean size of detected leaks (Figure 2b) have decreased, leading to a notable drop in detected emissions (Figure 2c). Well sites undergoing comprehensive surveys also show an overall decrease in detected emissions, where a small increase in the number of detected leaks (Figure 2a, noting the separate vertical scale for wells) is more than offset by a large decrease in mean detected leak size (Figure 2b). By contrast, screening surveys (AVO) are remarkably ineffective (Figure 2a), finding 21 times fewer sources than comprehensive surveys at well sites and finding no sources at 97% of sites. Although detected leaks via AVO were of similar magnitude when found (Figure 2b), the very low detection rate of screening surveys means they were ineffective at ascertaining emissions (Figure 2c).

**Figure 2:**
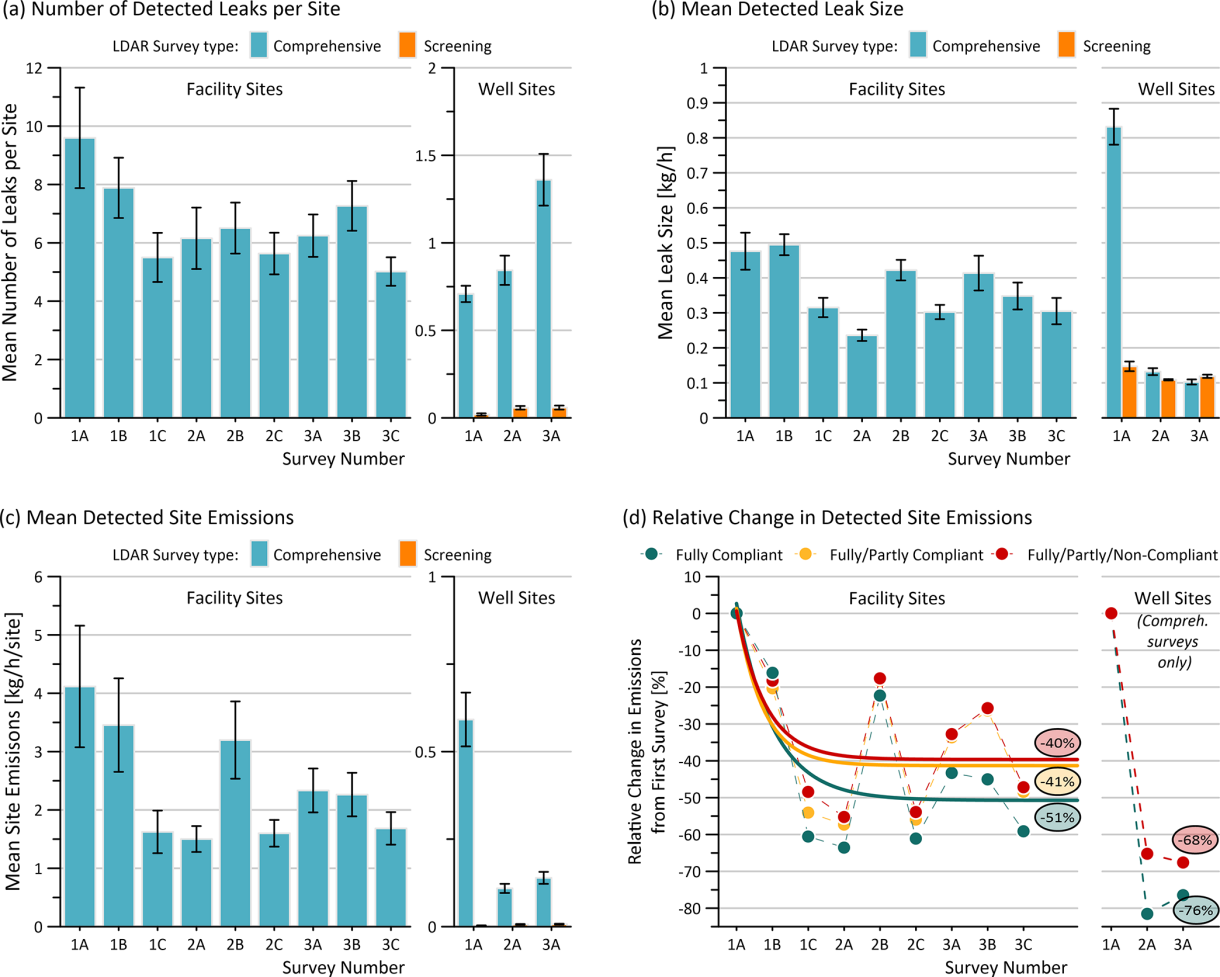
Trends in detected leaks during consecutive LDAR surveys at facility and well sites, where survey numbers 1A–1C correspond to the first, second, and third surveys in Year 1 (2020), 2A–2C in Year 2 (2021), etc. (a) Mean number of detected leaks per facility or well site via comprehensive or screening surveys at fully compliant sites. (b) Mean reported size of detected leaks. (c) Mean detected emissions per site for compliant Facility and Well Sites. (d) Mean percentage change in total detected site emissions relative to the first survey at facilities requiring 3×/yr comprehensive surveys and well sites requiring 1×/yr comprehensive surveys. Error bars represent the 95% confidence intervals in the mean.

Comparing facility sites and well sites that used comprehensive surveys, 85% of facility sites had detected emissions versus 38% of well sites. Moreover, facilities had ∼6 times more leaks per site, larger leak rates, and an order of magnitude more emissions per site. However, because there are roughly 10 times more well sites than facility sites, their total contribution to detected emissions is approximately the same. This underscores a key challenge in managing fugitive emissions: while mitigation strategies targeting high-emitting facilities can be efficient, the aggregate impact of many smaller sites can be equally or more important.

Figure 2d plots the change in detected total site emissions relative to the initial survey and fits exponential decay curves to the data. In general, the reported data suggest a significant decrease over the first two surveys and relatively constant emissions thereafter. Given reported repair date data indicating that approximately 80% of leaks are repaired within an average of <2 months after detection (See SI Figure S4), this implies that the relatively constant detection rate after the first two surveys is largely driven by the appearance of new leaks or the reoccurrence of previously repaired leaks. Unrepaired leaks are expected to be a comparatively minor contributor, noting that it is possible that the 20% of leaks without reported repair dates may have been repaired without subsequently reporting a repair date, left unrepaired and detected again, or left unrepaired and missed in a subsequent survey. Unfortunately, the lack of persistent leak identifiers in the available LDAR data means it is not possible to distinguish these possibilities. We recommend that regulators consider adding a persistent identifier (e.g., existing serial number or assigned label) to required reporting. This would permit tracking of recurrence rates and sustained repair effectiveness across repeated surveys, ultimately enabling prioritized inspections and maintenance. Notably, Figures 2 and S4 suggest that continued frequent surveys are essential for the long-term management of emissions.

For fully compliant facilities, 3×/yr comprehensive LDAR surveys appear to reduce *detected* emission sources by 51% in practice. This is about three-quarters of the 67.7% reduction predicted in simulations.^21^ However, because of imperfect regulatory compliance, the actual achieved reductions across all sites are somewhat lower. As elaborated in SI Section S2, the impact of missing surveys was estimated by assuming that emissions from a subsequent survey (when available) would have been present at the time of the prior missed survey. In cases where there was no subsequent survey, the population-average site emission rate for the first missed survey was applied. This analysis suggests that as currently implemented, the 3×/yr LDAR program is achieving an overall 40% reduction in detected emissions. Although it is not possible to fit a meaningful emissions decay curve at well sites with only three total surveys, the available data suggest similarly large reductions in detected emissions via comprehensive surveys.

Critically, however, the apparent reduction in detected emissions does not necessarily equate to an actual reduction in overall emissions. This is because LDAR surveys of fugitive sources using OGI cameras target and capture only a small portion of total emissions^27,28,30,37^ and the data in Figure 2 reflect changes in *detected* sources only. There will inevitably be leaks that are missed in any survey and hence go unreported. Additionally, there may be an element of “survey fatigue”, where the thoroughness of surveys declines over time, especially when conducted by the same internal teams.

### 3.3 Contrast in Measured Methane Sources During LDAR and Aerial Surveys

To better understand what portion of sources is being captured by regulated LDAR surveys, reported 2021 LDAR data from a set of 326 sites (encompassing 386 active facilities and 815 active wells) that exclusively completed comprehensive surveys were compared with independent source-resolved aerial measurements at the same sites collected during September 11 to October 8, 2021.^2^ Data for facilities with repeated LDAR surveys during 2021 were averaged and aggregated to permit a robust comparison. This approach is warranted given the lack of any trend in number of leaks per site, mean leak size, or mean detected site emissions for consecutive comprehensive LDAR surveys 2A–2C (Figure 2), and across all surveys, the consistent percentage of survey reports with detected leaks and percentage of leaks repaired (Figure S4).

As highlighted in Figure 3a, the aerial surveys found 12 times more methane emissions than comprehensive LDAR surveys at the same sites. This stark difference is consistent with Schwietzke et al.,^27^ who, using a different aerial technique, found 3–26 times more CH_4_ emissions than ground teams at a set of 257 sites in the Fayetteville Shale, and with Tyner and Johnson^28^ who found 18 times greater emissions when comparing aerial measurements with OGI survey data from one year prior at a sample of 140 sites in BC.

**Figure 3:**
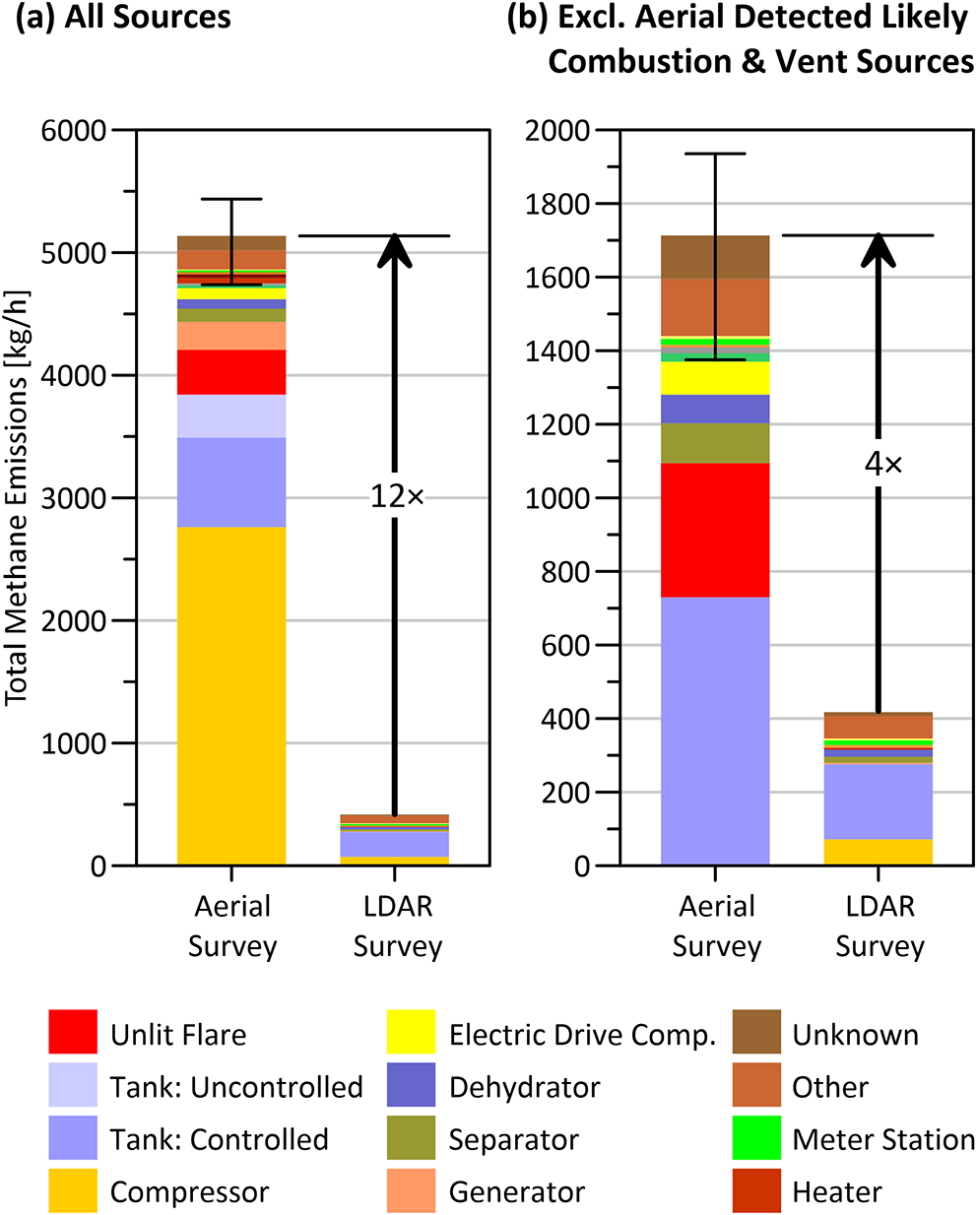
Contrast in CH_4_ emissions measured via comprehensive LDAR surveys and aerial gas mapping LiDAR (GML 1.0) at the same set of 326 sites (pads). (a) Comparison of all sources detected with each technology (b) Comparison of all detected LDAR sources with a subset of aerial detected sources excluding potential combustion sources (compressors, heaters, generators, lit flares) and venting sources (uncontrolled tanks, vent stacks) that are not expected to be captured in LDAR surveys. The error bars represent the 95% confidence interval on the total of the sample.

Noting that the scope of LDAR surveys is limited to equipment and components that may be sources of fugitive emissions,^36^ Figure 3b repeats the comparison excluding all aerially detected sources that could be combustion-related or classed as intentional vent sources making them unlikely to be seen or ignored by design in fugitive emission focused LDAR surveys. Specifically, Figure 3b conservatively excludes all aerial detections attributed to gas-fired compressors, generators, lit flares, and heaters under the assumption that they are dominated by methane slip within hot exhaust products, even though the reported LDAR data lists compressors, heaters, generators, and flare systems among the types of equipment associated with detected sources. Additionally, Figure 3b excludes uncontrolled tank sources and vent stacks from the aerial data. Even in this more conservative comparison, there is still a factor of 4 difference in detected emissions that is well beyond the variability among different LDAR surveys and well outside the estimated uncertainty limits of the aerial measurements. Figure S6 and Table S10 also show that these gaps persist across different equipment types and flights. Comparisons between aerial measurements and reported LDAR data from screening surveys shows even greater differences (SI, Figure S7).

These important results demonstrate that regulated LDAR surveys only capture a small portion of total methane emissions in practice. Conversely, the LDAR surveys detected more than twice as many sources than the aerial GML surveys at the same set of sites (Figure S8). Curiously, with the exception of uncontrolled tanks only seen in the aerial surveys, the equipment associated with detected sources is broadly similar, with both surveys identifying compressors, controlled tanks, and separators as the most common equipment (process blocks) associated with detected sources (Figures 3 and S9).

However, there are clear differences in the characteristics of the sources each technology finds. Whereas the aerial surveys found an average of 1.4 sources per site with a mean methane emission rate of 11 kg/h, comprehensive LDAR surveys found 3.9 sources per site (2.7x more) but with a mean emission rate of only 0.34 kg/h (33 times less, Figure S8). Fewer aerial detected sources is expected given that the first generation aerial GML technology used in this work has a 90% probability of detection (POD) of 1.7–5.8 kg/h at typical wind speeds^38^ versus 0.13 kg/h expected for an OGI camera used by an experienced operator in controlled conditions.^33^ Indeed, 86% of the sources detected during the LDAR surveys were estimated to be smaller than 0.5 kg/h and 46% of leaks were located inside buildings. Moreover, the leaks found within buildings were approximately three times smaller (as reported) than those identified outside, which would be challenging to detect via aerial measurements, especially if escaping the building as a diffuse plume.

What was not expected is that the LDAR surveys consistently failed to find the larger sources detected from the air. For example, although unlit flares accounted for 6% of the total measured provincial inventory in 2021,^2^ only 5 were reported as detected during the 2021 LDAR surveys and only 8 across all surveys from 2020–2022. Given that BC’s fugitive emissions management guidelines explicitly state that “*equipment used to combust vent gas*, *including burners*, *flare ignitors*, *and pilots”* must be surveyed,^36^ this suggests that the current LDAR program is failing to reliably detect unlit flares in practice.

Figure 4 examines this gap in greater detail and plots the distributions of reported emission rates for detected sources across all surveys (Figure 4a), and separately for sources linked with specific equipment or process blocks (Figure 4b–f), colored by the reported measurement approach. The LDAR and aerial source rate distributions are surprisingly distinct overall, and notably shifted for compressors, generators, and separators.

**Figure 4:**
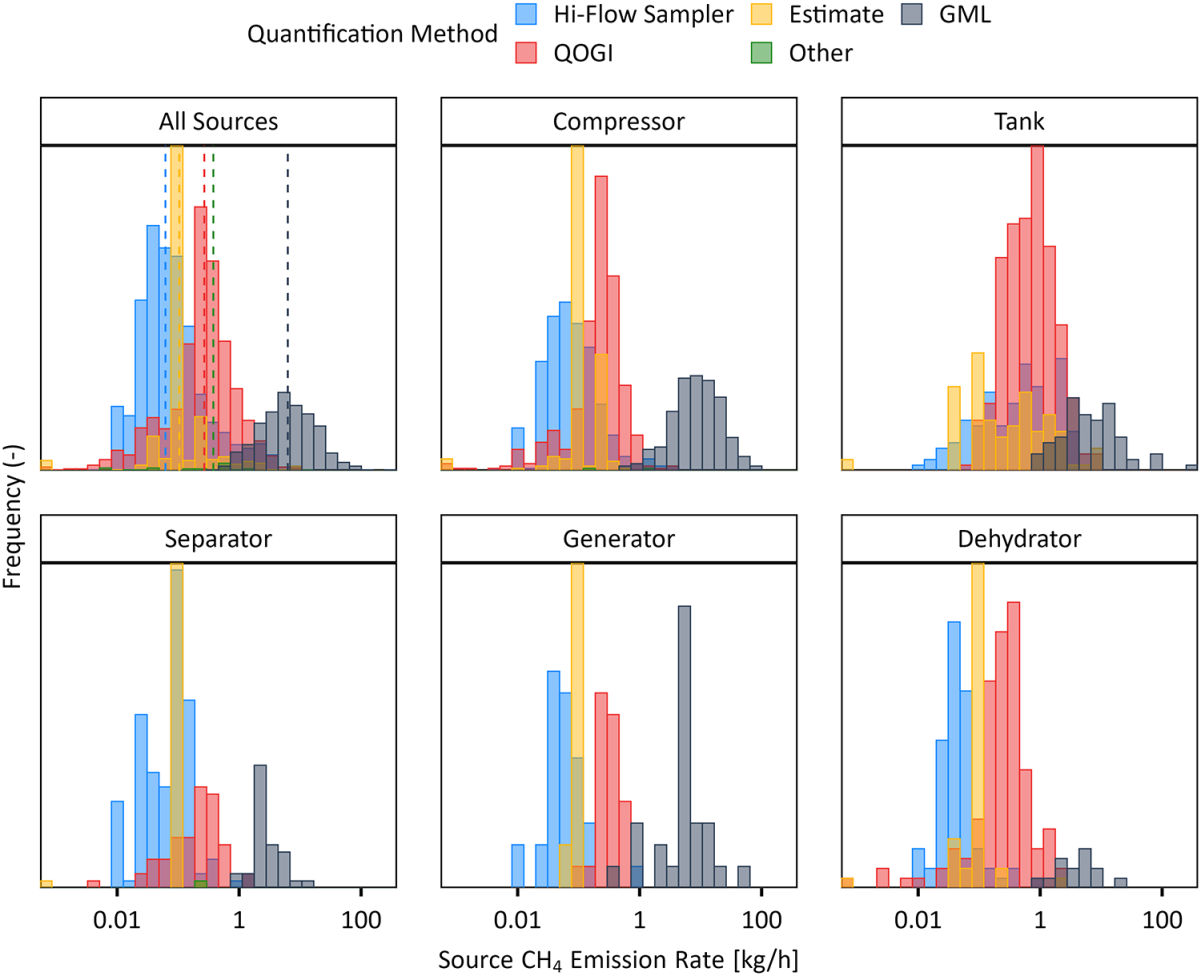
Methane leak rate distributions for the various quantification methods used in LDAR surveys, compared to GML data from the aerial survey. The dashed vertical lines in panel (a) indicate the median leak rate for each method. Note the log scale on the *x*-axis.

One possible reason for this difference, is that the reported rates in the LDAR surveys (which are often only estimated as permitted under current regulations) are inaccurate. Indeed, the distribution for estimated sources (yellow) reveal how the exact same estimated source rate (0.104 kg/h) unrealistically appears repeatedly in the data. Conversely, the distribution of estimated rates overlaps with the center of the distributions of Hi-Flow and QOGI measurements in all cases suggesting the estimates may nevertheless be reasonable. Although sources listed as measured using QOGI (red) have somewhat higher reported flow rates, they still generally fall below the levels in the aerial source distribution. Again, this is not necessarily surprising. Several recent studies have highlighted the inaccuracy of QOGI,^44–46^ reporting relative errors of −90% to +831% when measuring sources between 0.1 and 2.9 kg/h and “potential quantification challenges” at windspeeds above 4.5 m/s.^46^ Most notably, in a recent field study of tank venting,^45^ QOGI was incapable of responding to sources >10 kg/h, leading to consistent, gross underestimates of the separately metered vent rates. There are also technical limits of OGI cameras in real-world environments, where the lower detection limit goes to infinity (i.e., the ability to detect plumes goes to zero) in the absence of a temperature difference between the leak plume and the background.^47^ Similarly, multiple studies have reported that the Bacharach Hi-Flow Sampler system underestimates methane emission rates, particularly for VOC-rich leaks and poorly diluted airflow.^48,49^ Underestimates by up to 2 orders of magnitude can occur due to sensor transition failure, where the system fails to switch from a catalytic oxidation sensor used to measure low CH_4_ (∼5% or less) concentrations to a thermal conductivity sensor required for higher concentrations (from ∼5% to 100%).^50,51^ More generally, the Hi-Flow Sampler relies on the assumption that the emission source is completely entrained by the flow stream entering the sampler. In practice, a leak can be significantly underestimated if this is not true, which is more likely when access to the source is limited or restricted, as is the case for elevated sources such as thief hatches or pressure relief valves situated on tank tops.

Another possibility is that the aerial GML is overestimating source rates. However, this is unlikely given multiple studies demonstrating the ability of the GML to quantify sources within defined uncertainties both in fully- and semiblinded controlled release experiments^38,39,52^ and in comparative, in situ field measurements of separately metered sources.^45^ Most importantly, the plotted error bars in Figure 3, calculated via Monte Carlo analysis using the uncertainty model specific to the first generation GML technology used in the flights developed through blinded testing, is expected to encompass the true source total within 95% confidence. Similarly, the difference is not explained by potentially intermittent sources somehow seen only by the plane. Additional analysis shown in Figure S5 shows that the large differences remain whether considering only data from the initial aerial flights, only data from the subsequent revisit flights on a different day, or both together as presented in Figure 3. Most notably, in a parallel study using the same aerial data set,^37^ ground crews deployed 1–15 days after the flights confirmed detections and successfully attributed origins for 192 of 195 investigated aerially detected sources.

A third possibility is that the LDAR surveys are simply finding different sources than the aerial survey. Figure 5 suggests this may be the dominant factor. In addition to the above-noted observation that nearly half of LDAR sources were found inside buildings, 82% of all sources found in the LDAR surveys are connectors, valves, or other (where “other” includes leaking regulators, meters, and sources not specified in the available LDAR reports). These types of sources are generally too small to be seen in aerial surveys with a 90% probability of detection above 1.5 kg/h. For compressors, where the source rate distributions between the LDAR and aerial surveys are most distinct (Figure 4), 95% of reported LDAR sources are connectors, valves, or other. At these locations, the aerial detections were predominantly attributed to methane slip in the engine exhaust,^37^ which is not seen in OGI surveys but generally dominates overall compressor package emissions.^53^ Connectors, valves, and other also emerge as the most commonly detected LDAR sources associated with generators, dehydrators, and separators (see Figure S11). Consistent with the predominantly nonoverlapping LDAR and aerial source distributions (Figure 4), this suggests that for these types of equipment the two survey approaches are finding complementary subsets of sources.

**Figure 5:**
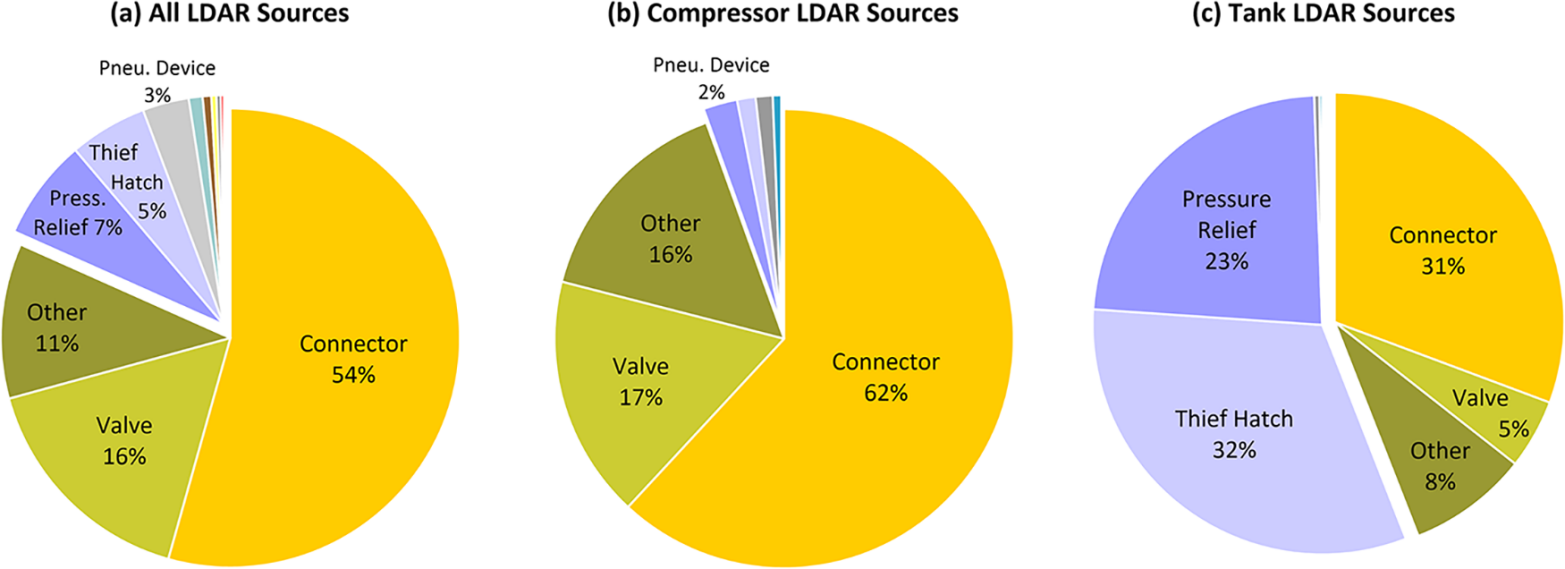
Breakdown of emitting sources detected in LDAR surveys: (a) all LDAR sources (*n* = 3302), (b) LDAR sources associated with compressors (*n* = 1000), and (c) LDAR sources associated with tanks (*n* = 531). Sources classed as other include leaking regulators, meters, and sources not specified in the available LDAR reports. See Figure S11 for breakdowns for additional equipment types.

By contrast, for tank related sources, thief hatches and pressure relief valves form the majority of LDAR detections, with less than half of sources attributed to connectors, valves, and other (Figure 5). The corresponding source rate distributions for tanks (Figure 4c) also show the greatest amount of overlap with aerial measurements. This implies that, at least for tanks, there are common sources detected by both survey methods. Indeed, thief hatches and pressure relief valves (PRV) were identified as the primary point of emission in follow-up inspections of aerially detected controlled tank sources^37^ and accounted for 70% of the total reported CH_4_ emissions from tanks in the LDAR surveys. However, there are still large discrepancies in the observed emission rates. The mean reported leak rate from thief hatches and PRVs in the LDAR survey (1.3 kg/h) was still an order of magnitude less than the mean emission rate of 17 kg/h attributed to controlled tanks in the aerial surveys. Pressure relief devices and thief hatches are typically located on tank tops, which are ideally placed for detection and quantification with the aerial technology but can pose visual access challenges for OGI detection from the ground. In addition, physical access challenges can complicate full plume capture for Hi-Flow sampler measurements, exacerbating the propensity for underestimation as discussed above. While survey guidelines stipulate that “technicians must use survey techniques that allow them to safely and effectively detect leaks at storage tank components”, and further that when using OGI “if there is a reasonable line of sight to a component, it must be surveyed”, they also state that components deemed “unsafe, difficult to survey, or inaccessible to survey....do not need to be included until it becomes feasible to do so”.^36^

## 4 Implications

The present analysis suggests that while comprehensive LDAR surveys are effective at reducing *detected* emissions, they only capture a small portion of total emissions in practice and thus should only represent one component of an overall effort to mitigate methane. This has critical implications for development of alternative strategies to detect, measure, and mitigate oil and gas sector methane sources, where regulatory acceptance of new approaches is often predicated on achieving equivalent performance to camera-based LDAR programs^54,55^ for which real-world performance has likely been overestimated. In the worst case, this could impede adoption of technologies or approaches with greater potential to efficiently identify and rapidly reduce methane sources. The analysis also shows that screening surveys using auditory, visual, olfactory (AVO) methods are wholly ineffective, and should not be considered an effective substitute for comprehensive surveys, as also noted previously.^34^

Conversely, this same analysis demonstrates how LDAR surveys can be a valuable complement to an alternative technology with a higher nominal detection threshold, largely finding a different subset of sources that combine to capture the full distribution of emissions. This underscores the utility of on-site camera surveys in investigating sources detected by other means^37^ or as part of a program for aerially guided leak detection and repair.^26,27^ Critically, however, the stark differences in both sources and magnitudes also highlight how emissions reconciliation programs such as OGMP 2.0 ^56^ should not be viewed as checking one technology against another, but rather, should be treated as an opportunity to combine two or more incomplete and imperfect sets of information to get a more complete and accurate understanding of total emissions and sources. Furthermore, while combined measurement approaches have clear advantages, the present example illustrates how different techniques are unlikely to be equivalent or equally impactful. In the present case specifically, aerial measurements like the ones deployed here appear to be much more valuable in identifying and mitigating primary drivers of emissions than the current real-world implementation of LDAR. This is an important consideration for regulators and policymakers tasked with designing or approving alternate LDAR strategies. Additional real-world, in situ intercomparisons of additional techniques would be extremely valuable in this regard. Finally, the results of this investigation demonstrate the importance of independent measurements in ensuring complete and accurate quantification of emissions. This verification is the core component of international measurement, reporting, and verification (MRV) efforts via OGMP2.0 ^56^ and is essential for driving rapid, effective, and efficient action to reduce emissions.

## Supplementary Material



## Data Availability

Data to replicate all figures in the main text and additional figures in the Supporting Information can be accessed via the Carleton University dataverse at: 10.5683/SP3/YO37TN. Site-anonymized, aerially measured, Monte Carlo- averaged emissions for all attributed sources in the 2021 survey are available at: https://pubs.acs.org/doi/10.1021/acs.est.2c07318.

## Notes

Determining rates of compliance (S1); calculating the change in emissions over time considering the level of compliance (S2); additional figures of analyzed reported LDAR data (S3); and references (S4) (PDF)
