# Supplementary material for: The Efficacy of Methane Leak Detection and Repair (LDAR) Programs in Practice
Source: ACS EST Air. 2025 Oct 28;2(11):2527–36. doi: 10.1021/acsestair.5c00195 (PMC12624710; doi:10.1021/acsestair.5c00195)
Supplement: Supplementary file 1 [file ea5c00195_si_001.pdf]

Supporting Information

The Efficacy of Methane Leak Detection and Repair (LDAR) Programs in Practice

Shona E. Wilde, David R. Tyner, Matthew R. Johnson*

Energy & Emissions Research Laboratory, Department of Mechanical and Aerospace Engineering, Carleton University, Ottawa, ON, Canada, K1S 5B6

*To whom correspondence and material requests should be addressed: Matthew.Johnson@carleton.ca; +1-613-520-2600 ext.4039.

File contains 25 pages, 11 figures, 10 tables

Table of Contents

S1 Determining Rates of Compliance 2

S1.1 Determination of Active Facilities and Wells 3

Initial Estimates of Active Counts from BC Facility Lists 3

Determining Active Status Leveraging Petrinex Production Data 4

S1.2 Determining the Required Number of LDAR Surveys 6

S1.3 Identifying Surveyed Facilities and Wells Co-located on the same Site (Pad) 10

Summary Compliance Data After Accounting for Co-Located Facilities and Wells 10

S1.4 External Government Review of Non-Compliant Facilities........ 12

S1.5 Compliance Based on Facility or Well Sites (Pads) 14

S2 Calculating the Change in Emissions Over Time Considering the Level of Compliance 17

S3 Additional Figures of Analyzed Reported LDAR Data 18

S4 References........ 24

S1 Determining Rates of Compliance

The purpose of this SI section is to provide exhaustive detail of how facilities and wells were determined to be active, and hence how compliance rates were calculated. Tables throughout the section are included to explain the steps in the process and how different (less accurate) compliance estimates might be obtained from simpler calculations. As an overview:

- Section S1.1 and Table S1 present initial raw facility counts based on current inaccurate public active facility and well lists and associated estimated compliance rates as a starting point.
- The remainder of Section S1.1 explains how separate volumetric reporting data were used to derive updated active facility and well counts and revised compliance estimates as summarized in Table S2.
- Section S1.2 explains how the number of required surveys was determined, including prorated requirements for facilities and wells that were operational during only part of the year. This, leads to the refined compliance data presented in Table S4 and Table S5, which assume that each facility or well must submit its own LDAR report as outlined in the BCER Fugitive Emissions Management Guideline (BCER, 2019) even if these facilities and wells are co-located on the same site (pad).
- Section S1.3 explains how facilities and well co-located on the same site (pad) were considered given other text in the same regulations stating that "(i)t is expected that all co-located wellsites and facilities undergo leak detection surveys whenever any of them is surveyed" (BCER, 2019). Table S6 and Table S7 then present summary compliance data allowing for the fact that surveys may only be recorded for a subset of the KERMIT IDs and WA numbers when in fact all infrastructure on the pad was surveyed.
- Section S1.4 summarizes results of an external Government review of facilities initially deemed non-compliant and Table S8 presents revised compliance data based on associated updated active facility counts.
- Finally, Section S1.5 and Table S9 and Figure S3 detail the final derived compliance rates considering data from Table S8 and calculated on a per pad basis as presented in the main text.

The British Columbia Drilling and Production Regulations (DPR) (BCOGC, 2021) states that comprehensive or screening surveys for fugitive emissions are required at facilities and wells with active equipment (e.g. pressurized in part or in whole) (BCER, 2019). Robust compliance statistics are therefore reliant on the accurate identification of active facilities and wells. Unfortunately, this is complicated by the fact that BC has two separate reporting systems (https://www.bc-er.ca/energy-professionals/online-systems):

- KERMIT, which "manages the operational lifecycle of pipelines and facilities including operational compliance and decommissioning", and
- Petrinex, which is used for volumetric reporting.

Additionally, the way in which facilities and wells are identified is not consistent between the two systems, such that a single KERMIT facility may correspond to one or more Petrinex facilities and vice versa. As further discussed below, there may also be multiple KERMIT- or Petrinex-defined facilities within a single site or pad, which raises the possibility that an LDAR survey may be completed at the site but not necessarily separately reported for each of the facilities on that site.

The regulatory guidelines for completing LDAR surveys require reporting based on the KERMIT system, i.e., completed survey data are to be reported under their relevant KERMIT facility identifier and/or well authorization (WA) number. Therefore, the rate of compliance must be assessed by comparing against an inventory of all active facilities and wells outlined in the same system. However, although a comprehensive list of all KERMIT facilities and wells in BC is accessible to the public through BCER, it is also generally accepted that the status of facilities (i.e., active, inactive, suspended, removed, etc.) in this list may not be accurate or may be delayed in being updated (BCER, 2021). To overcome this challenge, we also leveraged monthly volumetric data reported thought the Petrinex system to more definitively ascertain the active status of individual facilities and wells as elaborated below.

S1.1 Determination of Active Facilities and Wells

Initial Estimates of Active Counts from BC Facility Lists

A copy of the public BC KERMIT facility inventory file [BCOGC-41090](BCER, 2021) was first obtained (downloaded in October, 2023), which listed the facility name, facility type, facility location, current status (i.e. Active, Suspended, Removed), operational start date, suspended date, reactivated date, and last recorded production date. So-called "Well Facilities" within this Kermit inventory file were separately considered as individual wells, identified by their WA number as separately discussed below. Facilities other than "Well Facilities" were initially identified as being active throughout a given year (i.e., 2020, 2021, or 2022 in the present analysis) based on having a listed operational start date before, and a last production date after the specified year or a blank last production date, as summarized in Table S1.

Table S1: Initial raw counts of active facilities in the BC Facility Inventory File [BCOGC-41090], producing wells appearing in BC total production data [BCOGC-41143], and facilities or wells included in received LDAR reports.

	Number of KERMT Facilities			Fraction with at least one submitted LDAR Report [%]		
	2020	2021	2022	2020	2021	2022
**Total Active Facilities (excluding "Well Facilities") based on Kermit inventory file or received LDAR reports**	**2181**	**2099**	**2053**	**27**	**30**	**31**
Active throughout year	2052	2007	1938	26	28	29
Active during part of year	70	23	41	1	4	5
Additional facilities not in Active List but with submitted LDAR reports	59	69	74	100	100	100
Well Facilities	6303	6326	6349	0	16	14
**Total Active Wells based on BC production data or received LDAR reports (WA Numbers)**	**9947**	**10258**	**10649**	**59**	**85**	**89**
Wells linked to Well Facilities	7336	7719	7995	65	88	91
Additional wells not in Active List but with submitted LDAR reports	60	172	329	100	100	100
Wells not linked to a Well Facility (Off-site wells)	2551	2367	2325	42	76	78
Active Kermit Facilities Excluded from LDAR Regulations						
LNG Facilities	4	5	5	-	-	-
Pump Stations	19	20	20	-	-	-
"Paper Facilities"	80	77	73	-	-	-
Alberta Facilities Reporting via BC	21	20	20	-	-	-

Facilities that were either suspended or reactivated during the specified year, were initially considered as partially active, indicating the potential need for leak detection surveys at a prorated frequency as elaborated below. Facilities that are exempt from LDAR regulations were excluded from the study. These included Liquefied Natural Gas (LNG) facilities, pipeline equipment, and pipeline gathering facilities not subject to the DPR. Pump stations were also excluded because it was assumed they use diesel-driven engines to pump river water, and therefore methane emissions are not expected during their operation. Additionally, "Paper Facilities" (facility codes which are created for production accounting purposes and do not correspond to physical facilities (BCER, 2023a)) and facilities located in the neighboring province of Alberta that report to BCER, determined from the facility location represented by a Dominion Land Survey (DLS) or National Topographic System (NTS) identifier, were excluded. Finally, facilities, other than Well Facilities, that were included in the industry-reported LDAR survey data but were not present in the BC active facilities list, were incorporated. This resulted in an initial list of 2,105 unique facilities identified as active at some point between 2020 and 2022. Summary counts of the identified facilities included for each year are shown in Table S1. Of these presumably active facilities, only 27%, 30%, 31% were present in the LDAR data set for 2020, 2021 and 2022, respectively, indicating a potentially very low compliance with regulations. However, as shown below, this apparent poor compliance appears to be largely due to the (i) inaccuracies in the publicly available lists of active facility and wells, and (ii) the co-location of facilities and wells, where survey reporting may not be separate if there are multiple wells or facilities located on a single site.

Separately, a list of wells was compiled from BC Total Production data [BCOGC-41143] (BCER, 2023b). Any well (defined by a WA) where the total gas production during each year was greater than zero was included as active. Wells that were included in the industry-reported LDAR survey data but were not present in the BC active wells list were incorporated, comprising 9947, 10258 and 10649 wells (WA) in 2020, 2021 and 2022, respectively. Wells can either be part of a Well Facility —defined as "a facility associated with one or more wells that typically includes a simple piping and equipment configuration"— or be located separately without additional infrastructure, known as "off-site" wells. LDAR reports are submitted according to the KERMIT ID for facilities and the WA number for wells. However, this is more complex for Well Facilities, where an LDAR report can be submitted using *either* the Facility ID of the Well Facility or the WA number of each surveyed well. In the present analysis, an LDAR report submitted for a KERMIT Well Facility was assumed to include all wells (WA) associated with that facility.

Determining Active Status Leveraging Petrinex Production Data

To more reliably determine the operational status of facilities and wells, their monthly production volumes reported via the Petrinex system were also considered. For facilities and wells appearing in Petrinex there are 22 associated activity codes (Petrinex, 2022) in the monthly volumetric data which track production, flaring, venting, transfers (receipts and dispositions), storage (injection), and losses (shrinkage and metering differences) of produced and processed fluid volumes. In the present analysis, a facility or well was deemed active in any month when the volume of gas, oil, condensate, or water pertaining to any one of the 22 activity codes was non-zero.

The production volumes for wells identified by a WA number can be directly extracted from the Petrinex volumetric data in a two-step process. First, the BCER "well index file" (BCER, 2023c) was used to link "unique well identifiers" (UWI, which label individual segments of a well within the Petrinex system) to their shared surface holes (wells), each identified by a well authorization number (WA). Next, the production volumes for all UWI (which is reported via linked facilities within Petrinex) associated with each WA were summed to give the totals for each well, and WA reporting any volumes in any given month were considered active.

For the 2,105 KERMIT facilities identified from the BC Facility Inventory file, production volumes were inferred by relating KERMIT facility IDs to Petrinex facility IDs. Because this correspondence is not one to one, a multistep approach was needed to derive the necessary linkages. An initial list of Petrinex facility IDs was compiled from the Petrinex volumetric data, which included any facility that reported activity during any month between 2020 and 2022. Then, where possible, KERMIT ID numbers were directly matched to the numerical portion of a corresponding Petrinex ID(s), noting that these associations can exist in one-to-many relationships. For example, KERMIT Battery Site 0000160 corresponds to three smaller facilities in Petrinex (BCBT0000160, BCGP0000160, BCIF0000160) at the same location. The accuracy of these inferred correspondences was separately checked by geolocation to confirm that the matched facilities were indeed all co-located on the same pad. Of the 391 Kermit IDs with matched Petrinex IDs, 382 were confirmed to be co-located and directly matched. The remaining 8 were assumed to be unrelated (e.g., KERMIT Battery Site 0000827 is 132 km away from Petrinex facility BCMS0000827 and thus not the same facility).

Next, facilities identified by Kermit IDs and those identified by Petrinex IDs were linked if they were geolocated in the same physical well pad / production site. From careful manual inspection of georeferenced satellite and aerial imagery, 1,710 polygons defining pad boundaries were created that encompassed the 2,105 unique Kermit facilities (excluding Well Facilities) from Table S1. A further, 456, 435, and 434 Kermit-identified facilities were then deemed active in 2020, 2021, and 2022, respectively, based on the existence of a verified active Petrinex-identified facility (based on production volumes) within the same pad. For 110 Kermit Facilities identified as Compressor Stations and Compressor Dehydrators that were co-located in a pad with Petrinex-identified compressors stations (Petrinex facility subtype 601) and no other Petrinex facility types, the active status of these Kermit facilities could not be verified. This is because under current Petrinex reporting rules, compressor stations (subtype 601) do not separately report volumetric data or fuel use. Thus, these 110 Kermit-identified compressor facilities were necessarily assumed to be active based on the available data in the BC Facility Inventory File. An additional 157, 206, and 210 facilities in 2020, 2021 and 2022, respectively, which were not co-located with an active Petrinex facility were deemed active based on the existence of at least one LDAR report.

Finaly, there remained 1068, 1003, and 981 KERMIT facilities that could not be matched with any Petrinex facilities in 2020, 2021, and 2022 and were also not present in the LDAR data set. These facilities were assumed to be inactive. In a few cases, this assumption was confirmed via visual inspection of available satellite or aerial imagery which showed facilities as no longer existing. The breakdown of the revised list of active facilities (leveraging reported production data) and corresponding received LDAR reports is summarized in Table S2.

Table S2: Revised counts of active KERMIT facilities and wells by type (filtered to only include facilities or wells with reported volumetric data where those entities report via Petrinex) and corresponding fractions of facilities and wells with at least one submitted LDAR survey

	Total Active or Partially Active			Number with at least one submitted LDAR Report			Fraction with at least one submitted LDAR report [%]		
**KERMIT Facility Type**	**2020**	**2021**	**2022**	**2020**	**2021**	**2022**	**2020**	**2021**	**2022**
Battery Site	119	105	94	67	67	64	56	64	68
Compressor Dehydrator	163	151	146	116	121	116	71	80	79
Compressor Station	119	120	116	51	64	62	43	53	53
Disposal Station	44	44	43	28	29	29	64	66	67
Gas Dehydrator	11	9	10	5	4	6	45	44	60
Gas Processing Plant	77	76	75	68	70	69	88	92	92
Gas Sales Meter	197	186	180	44	40	39	22	22	22
Injection Station	7	6	6	4	3	3	57	50	50
NGL Fractionation Facility	1	1	1	1	1	1	100	100	100
Oil Sales Meter	68	59	58	42	17	18	62	29	31
Pipeline Gathering	2	1	1	0	0	0	0	0	0
Processing Battery	48	46	45	35	35	36	73	76	80
Satellite Battery	107	140	149	97	132	143	91	94	96
Tank Terminal	9	9	9	8	8	8	89	89	89
Water Hub	15	18	17	4	6	7	27	33	41
Well Facility	4962	4849	4816	3246	4237	4327	65	87	90
**Total Active Facilities (Excluding "Well Facilities")**	**987**	**971**	**950**	**570**	**597**	**601**	**58**	**61**	**63**
**Total Active Wells (WA Number)***	**9947**	**10258**	**10649**	**5869**	**8752**	**9451**	**59**	**85**	**89**
**Total *(counting wells individually rather than as `Well Facilities`)***	**10934**	**11229**	**11599**	**6439**	**9349**	**10052**	**59**	**83**	**87**

* Considered active if WA number appears in Petrinex and considered surveyed if either the WA has a corresponding LDAR report or if the WA number is linked to a Well Facility that has an LDAR report

S1.2 Determining the Required Number of LDAR Surveys

Section 41.1 of the DPR specifies required survey types and frequencies, which vary according to facility or well type, production type, and by the presence or absence of storage tanks. "Comprehensive surveys", using either an optical gas imaging (OGI) camera or an organic vapour analyzer capable of detecting natural gas at 500 ppm, are required:

- three times per year at gas processing plants, compressor stations, multi-well batteries, and single-well batteries with a controlled storage tank,
- one time per year at custom treating facilities, disposal or injection facilities, all other single well batteries, and all other facilities with storage tanks, and
- one time per year for wells with a storage tank or wells that produce from an unconventional zone.

For any other case, only an annual "screening survey" is required, in which leaks are detected qualitatively using human senses of hearing, sight, and smell, or via a soap bubble test.

Determining the number and type of required surveys was thus a multi-step process. First, using listed KERMIT facility types, compressor stations, compressor dehydrators, and gas processing plants were identified as each requiring 3 comprehensive surveys per year, whereas custom treating facilities, disposal facilities, and injection facilities were identified as requiring only 1 annual comprehensive survey. The BCER KERMIT data lacks facility types titled "multi-well battery" (MWB) or "single-well battery" (SWB) as these are Petrinex sub types (Petrinex, 2023). Therefore, the relevant classification of KERMIT batteries (facility types: Battery Site, Satellite Battery, Processing Battery) was determined by again relating KERMIT facility IDs to Petrinex facility IDs as in Section 1.1. Multi-well and single-well batteries were defined according to Petrinex sub-types as follows:

- Multi-well batteries (MWB): Facility sub-types 321, 322, 361, 362, 364, 365, 393, 401, 402, 403, 404, 405, 406 and 407
- Single-well batteries (SWB): Facility sub-types 311, 331, 351, and 902

In cases where a KERMIT battery site could not be linked to corresponding Petrinex multi- or single-well battery, we conservatively assumed these were single-well batteries and hence only one comprehensive survey was required. For SWB, the required number of surveys depends on the presence of *controlled* storage tanks which is not separately tracked by the government. Thus, the analysis assumes that SWB do not typically include *controlled* storage tanks and conservatively assumes that only one comprehensive survey per year is required at SWB sites. We also conservatively assume that remaining facility types (i.e., water hubs and sales meters) do not include storage tanks, and consequently only require one screening survey per year.

For wells, for which only a single annual survey is required, the survey type (comprehensive or screening) depends on whether the well is located within the unconventional production zones defined in Schedule 2 of the DRP (BCOGC, 2021) and available as a shapefile [BCOGC-44808] (BCER, 2023d). Wells within these zones require one comprehensive survey per year, whereas wells outside these zones only require an annual screening survey (under the conservative assumption that no storage tanks were present at these wells).

For partially active facilities and wells, the number of required surveys was based on the number of active months, determined from Petrinex volumetric data, and the proration tables within the Fugitive Emissions Management Guideline (BCER, 2019), reproduced here as Table S3. In cases where a single KERMIT facility was represented by multiple Petrinex entities, the number of required surveys was based on the entity with the largest number of active months. For the additional facilities listed in the LDAR reports which could not be linked with a Petrinex facility, the count of pressurized days, specified within the LDAR data, was used to determine the active number of active months during each year. For compressor stations and compressor dehydrators not linked to a Petrinex facility or in the LDAR reports, the assumption was made that the number of active months was twelve, given the lack of Petrinex volumetric data to verify activity. Summaries of the estimated compliance split by survey type and facility type are provided in Table S4 and Table S5. These compliance statistics assume that each facility, Well Facility, or well (WA)

must submit its own LDAR report as outlined in the BCER Fugitive Emissions Management Guideline (BCER, 2019). Section S1.3 repeats this analysis considering that co-located facilities may be reported in a single report for that site.

Table S3: BCER prorated comprehensive survey requirements for sites that were partially active during the year

Days Active (Pressurized) per Calendar Year	Number of Comprehensive Surveys Required per Calendar Year
For sites that would be subject to three comprehensive surveys if fully active	
0-30	0
31-121	1
122-243	2
244-365	3
For sites that would be subject to one comprehensive surveys if fully active	
0-90	0
91-365	1

Table S4: Compliance determined for active facilities and wells listed in Table S2 split by the type (comprehensive or screening) and number of required surveys.

Year	Required Survey Type	*Facilities*						*Wells*	
		Number Active during >30 days of year*	Number Requiring Surveys at:			Fraction with at least one Survey	Fully Compliant Fraction (with ALL required surveys)	Number Active >90 days per year, requiring 1×/yr surveys*	Fully Compliant Fraction (fraction surveyed)
			3×/yr	2×/yr	1×/yr				
**2020**	Comprehensive	534	351	17	166	62	49	6212	66
	Screening	275	-	-	275	29	29	2950	50
**2021**	Comprehensive	492	331	5	156	68	58	6714	92
	Screening	250	-	-	250	22	22	2918	76
**2022**	Comprehensive	474	331	2	141	69	65	7194	94
	Screening	250	-	-	250	24	24	2985	81

* Facilities active (pressurized) more than 30 days per year and wells active more than 90 days per year are subject to LDAR regulations at prorated survey frequency as per Table S3.

Table S5: Summary LDAR survey compliance data *assuming individually submitted reports* (i.e., that each facility or well (WA) must submit its own LDAR report as outlined in the BCER Fugitive Emissions Management Guideline (BCER, 2019))

KERMT Facility Type	Number Active during All or Some Months			Comprehensive Surveys									Screening Surveys					
				Number Requiring one or more Comprehensive LDAR Surveys*			Fraction of Facilities and Wells with at least one LDAR Survey [%]			Fraction of Fully Compliant Facilities or Wells [%]			Number Requiring Screening LDAR Survey*s			Fraction of Fully Compliant Facilities or Wells [%]		
	2020	2021	2022	2020	2021	2022	2020	2021	2022	2020	2021	2022	2020	2021	2022	2020	2021	2022
Battery Site	119	105	94	99	85	76	53	61	67	46	58	63	n/a	n/a	n/a	n/a	n/a	n/a
Compressor Dehydrator	163	151	146	152	142	134	72	80	78	52	65	75	n/a	n/a	n/a	n/a	n/a	n/a
Compressor Station	119	120	116	103	93	91	35	42	42	20	30	35	n/a	n/a	n/a	n/a	n/a	n/a
Disposal Station	44	44	43	37	37	36	62	62	64	62	62	64	n/a	n/a	n/a	n/a	n/a	n/a
Gas Dehydrator	11	9	10	n/a	n/a	n/a	n/a	n/a	n/a	n/a	n/a	n/a	8	7	7	25	29	43
Gas Processing Plant	77	76	75	74	72	72	88	93	92	76	75	86	n/a	n/a	n/a	n/a	n/a	n/a
Gas Sales Meter	197	186	180	n/a	n/a	n/a	n/a	n/a	n/a	n/a	n/a	n/a	188	169	170	20	20	20
Injection Station	7	6	6	5	5	5	60	40	40	60	40	40	n/a	n/a	n/a	n/a	n/a	n/a
NGL Fractionation Facility	1	1	1	n/a	n/a	n/a	n/a	n/a	n/a	n/a	n/a	n/a	1	1	1	100	100	100
Oil Sales Meter	68	59	58	n/a	n/a	n/a	n/a	n/a	n/a	n/a	n/a	n/a	62	56	55	58	27	29
Pipeline Gathering	2	1	1	n/a	n/a	n/a	n/a	n/a	n/a	n/a	n/a	n/a	1	1	1	0	0	0
Processing Battery	48	46	45	43	38	39	70	74	77	60	74	74	n/a	n/a	n/a	n/a	n/a	n/a
Satellite Battery	107	140	149	14	13	13	36	38	54	21	38	46	n/a	n/a	n/a	n/a	n/a	n/a
Tank Terminal	9	9	9	7	7	8	86	86	88	86	86	88	n/a	n/a	n/a	n/a	n/a	n/a
Water Hub	15	18	17	n/a	n/a	n/a	n/a	n/a	n/a	n/a	n/a	n/a	15	16	16	27	25	38
Total Facilities	**987**	**971**	**950**	**534**	**492**	**474**	**62**	**68**	**69**	**49**	**58**	**65**	**275**	**250**	**250**	**29**	**22**	**24**
**Total Wells**	**9947**	**10258**	**10649**	**6212**	**6714**	**7194**	**66**	**92**	**94**	**66**	**92**	**94**	**2950**	**2918**	**2985**	**50**	**76**	**81**
**Total**	**10934**	**11229**	**11599**	**6746**	**7206**	**7668**	**65**	**90**	**92**	**64**	**89**	**92**	**3225**	**3168**	**3235**	**48**	**72**	**76**

* Facilities active (pressurized) more than 30 days per year and wells active more than 90 days per year are subject to LDAR regulations at prorated survey frequency as per Table S3.

S1.3 Identifying Surveyed Facilities and Wells Co-located on the same Site (Pad)

The Fugitive Emissions Management Guideline states that (BCER, 2019):

> *"It is expected that all co-located wellsites and facilities undergo leak detection surveys whenever any of them is surveyed. For example, if a wellsite is co-located with a multi-well battery, each time the multi-well battery is surveyed, the co-located wellsite would also be surveyed"*

Given that a single site, defined as all infrastructure located on a single pad, may contain multiple KERMIT facilities and/or wells, it is reasonable or likely that surveys may only be recorded for a subset of the KERMIT IDs and WA numbers when in fact all infrastructure was surveyed. Therefore, we further consider the possibility that any facility or well situated on the same pad as a facility or well which underwent at least one LDAR survey was itself also surveyed.

Facility boundaries for were defined by manually drawing geo-located polygons guided by satellite and/or high-resolution aerial imagery as elaborated in S1.1. A total of 5,835 polygons defining facility and well sites (including both single and multi-well pads) were created. The total number of submitted LDAR surveys for each site during each year was then determined, based on the requirement that the interval between required consecutive surveys in a single year must be at least 60 days (BCOGC, 2021). For each site, we counted the total number of submitted surveys from all KERMIT facilities and wells within each polygon that were separated by at least 60-day intervals within a given year. We then assumed that all active KERMIT facilities and wells within that site were included in all the surveys. Table S6 shows the compliance for active facilities and wells based on the required number and type of survey after accounting for co-location. This suggests substantially higher compliance rates than Table S4. A further breakdown of the compliance for individual facility types is presented in Table S7.

Summary Compliance Data After Accounting for Co-Located Facilities and Wells

Table S6: Compliance determined for active facilities and wells listed in Table S2 split by the type of survey considering that some co-located facilities or wells may report together.

<table>
<tr><th rowspan="3">Year</th><th rowspan="3">Required Survey Type</th><th colspan="6">Facilities</th><th colspan="2">Wells</th></tr>
<tr><th rowspan="2">Number Active during >30 days of year*</th><th colspan="3">Number Requiring Surveys at:</th><th rowspan="2">Fraction with at least one Survey</th><th rowspan="2">Fully Compliant Fraction (ALL required surveys)</th><th rowspan="2">Number Active >90 days per year, requiring 1×/yr surveys</th><th rowspan="2">Fully Compliant Fraction (fraction surveyed)</th></tr>
<tr><th>3×/yr</th><th>2×/yr</th><th>1×/yr</th></tr>
<tr><td rowspan="2">2020</td><td>Comprehensive</td><td>534</td><td>351</td><td>17</td><td>166</td><td>71</td><td>54</td><td>6212</td><td>78</td></tr>
<tr><td>Screening</td><td>275</td><td>-</td><td>-</td><td>275</td><td>58</td><td>58</td><td>2950</td><td>57</td></tr>
<tr><td rowspan="2">2021</td><td>Comprehensive</td><td>492</td><td>331</td><td>5</td><td>156</td><td>77</td><td>65</td><td>6714</td><td>95</td></tr>
<tr><td>Screening</td><td>250</td><td>-</td><td>-</td><td>250</td><td>67</td><td>67</td><td>2918</td><td>81</td></tr>
<tr><td rowspan="2">2022</td><td>Comprehensive</td><td>474</td><td>331</td><td>2</td><td>141</td><td>78</td><td>68</td><td>7194</td><td>95</td></tr>
<tr><td>Screening</td><td>250</td><td>-</td><td>-</td><td>250</td><td>65</td><td>65</td><td>2985</td><td>86</td></tr>
</table>

* Facilities active (pressurized) more than 30 days per year and wells active more than 90 days per year are subject to LDAR regulations at prorated survey frequency as per Table S3.

Table S7: Summary LDAR survey compliance data based on facility type considering that some co-located facilities or wells may report together.

KERMT Facility Type	Number Active during All or Some Months			Comprehensive Surveys									Screening Surveys					
				Number Requiring one or more Comprehensive LDAR Surveys*			Fraction of Facilities and Wells with at least one LDAR Survey [%]			Fraction of Fully Compliant Facilities or Wells [%]			Number Requiring Screening LDAR Surveys*			Fraction of Fully Compliant Facilities or Wells [%]		
	**2020**	**2021**	**2022**	**2020**	**2021**	**2022**	**2020**	**2021**	**2022**	**2020**	**2021**	**2022**	**2020**	**2021**	**2022**	**2020**	**2021**	**2022**
Battery Site	119	105	94	99	85	76	53	61	67	58	73	76	n/a	n/a	n/a	n/a	n/a	n/a
Compressor Dehydrator	163	151	146	152	142	134	72	80	78	54	63	72	n/a	n/a	n/a	n/a	n/a	n/a
Compressor Station	119	120	116	103	93	91	35	42	42	21	34	33	n/a	n/a	n/a	n/a	n/a	n/a
Disposal Station	44	44	43	37	37	36	62	62	64	76	76	69	n/a	n/a	n/a	n/a	n/a	n/a
Gas Dehydrator	11	9	10	n/a	n/a	n/a	n/a	n/a	n/a	n/a	n/a	n/a	8	7	7	50	71	71
Gas Processing Plant	77	76	75	74	72	72	88	93	92	77	81	89	n/a	n/a	n/a	n/a	n/a	n/a
Gas Sales Meter	197	186	180	n/a	n/a	n/a	n/a	n/a	n/a	n/a	n/a	n/a	188	169	170	53	59	57
Injection Station	7	6	6	5	5	5	60	40	40	80	80	80	n/a	n/a	n/a	n/a	n/a	n/a
NGL Fractionation Facility	1	1	1	n/a	n/a	n/a	n/a	n/a	n/a	n/a	n/a	n/a	1	1	1	100	100	100
Oil Sales Meter	68	59	58	n/a	n/a	n/a	n/a	n/a	n/a	n/a	n/a	n/a	62	56	55	76	89	89
Pipeline Gathering	2	1	1	n/a	n/a	n/a	n/a	n/a	n/a	n/a	n/a	n/a	1	1	1	100	100	100
Processing Battery	48	46	45	43	38	39	70	74	77	60	76	74	n/a	n/a	n/a	n/a	n/a	n/a
Satellite Battery	107	140	149	14	13	13	36	38	54	57	69	69	n/a	n/a	n/a	n/a	n/a	n/a
Tank Terminal	9	9	9	7	7	8	86	86	88	100	100	100	n/a	n/a	n/a	n/a	n/a	n/a
Water Hub	15	18	17	n/a	n/a	n/a	n/a	n/a	n/a	n/a	n/a	n/a	15	16	16	47	62	62
Total Facilities	**987**	**971**	**950**	**534**	**492**	**474**	**71**	**77**	**78**	**54**	**65**	**68**	**275**	**250**	**250**	**58**	**67**	**65**
Wells	9947	10258	10649	6212	6714	7194	78	95	95	78	95	95	2950	2918	2985	57	81	86
**Total**	**10934**	**11229**	**11599**	**6746**	**7206**	**7668**	**77**	**93**	**94**	**76**	**93**	**93**	**3225**	**3168**	**3235**	**57**	**80**	**85**

* Facilities active (pressurized) more than 30 days per year and wells active more than 90 days per year are subject to LDAR regulations at prorated survey frequency as per Table S3.

S1.4 External Government Review of Non-Compliant Facilities

The apparent low compliance rates for Compressor Stations in Table S7 is partially attributable to the inaccuracies in BC's active facility list, noting that because these facility types do not report in Petrinex, their activity status could not be separately verified using Petrinex volumetric data. To verify both the existence and activity status of these facilities, a further review of all apparently non-compliant facilities was conducted in collaboration with BCER.

An initial list of non-compliant facilities determined from Table S7 was shared with BCER for their review using additional data sets not available to the public. Firstly, BCER was able to verify that some facilities submitted the required number of LDAR surveys and were therefore compliant, though these data were not included in the online public dataset. Second, aerial imagery plus BCER inspection records were used to verify the presence and status of facilities during each year, revealing that some facilities were inactive or had been removed, but their status in the public data set had not yet been updated. Similarly, BCER identified orphaned facilities, which are no longer in operation and are not maintained by a company or operator. Facilities, where no natural gas was present, were identified by analyzing Petrinex volumetric data at the facility location. For instance, a facility, such as a Water Hub, that was initially thought to be active due to reported activity in Petrinex might be found to report only volumes of water or sand. This indicates that no natural gas was actually present at the site, and hence there would be no "pressurized days" such that LDAR surveys were not required. Similarly, Oil Sales Meters were assumed by BCER to handle treated oil and not expected to have any gas present unless co-located with another facility. BCER were also able to identify facilities where the number of production days did not meet the minimum threshold for survey requirements (Table S3). Finally, in a small number of cases, BCER contacted permit holders directly to confirm the status of facilities for the specified year.

Figure S1 shows the revised status of facilities after review by BCER. Following this review, it was determined that approximately 67%, 82%, and 80% of facilities initially considered non-compliant in 2020, 2021 and 2022, respectively, were actually inactive, removed, or did not require LDAR surveys. This reclassification led to a significant increase in the calculated compliance rates of some facility types, as detailed in Table S8 and plotted in Figure S2.

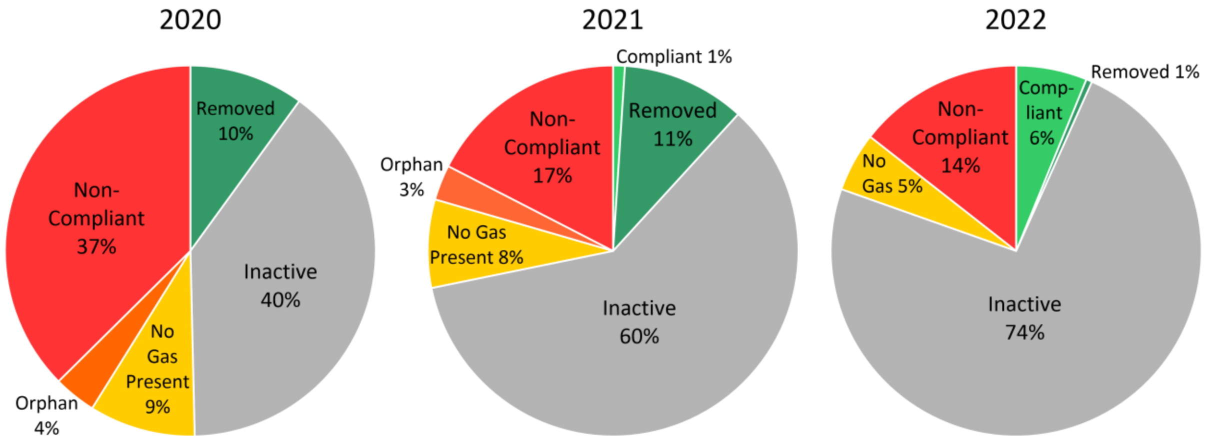

Figure S1: Revised status of facilities initially found to be non-compliant following an external review by BCER.

Table S8: Summary LDAR survey compliance data based on facility type after accounting for co-location as in Table S7, but with updated active counts following an external Government review of the facilities initially considered to be non-compliant.

KERMT Facility Type	Number Active during All or Some Months			Comprehensive Surveys									Screening Surveys					
				Number Requiring one or more Comprehensive LDAR Surveys*			Fraction of Facilities and Wells with at least one LDAR Survey [%]			Fraction of Fully Compliant Facilities or Wells [%]			Number Requiring Screening LDAR Surveys*			Fraction of Fully Compliant Facilities or Wells [%]		
	2020	2021	2022	2020	2021	2022	2020	2021	2022	2020	2021	2022	2020	2021	2022	2020	2021	2022
Battery Site	104	92	86	84	73	71	62	71	72	68	86	85	n/a	n/a	n/a	n/a	n/a	n/a
Compressor Dehydrator	149	131	126	138	122	114	80	93	92	59	74	87	n/a	n/a	n/a	n/a	n/a	n/a
Compressor Station	79	79	78	63	52	53	57	75	72	35	63	58	n/a	n/a	n/a	n/a	n/a	n/a
Disposal Station	34	33	31	27	26	25	74	85	92	93	96	100	n/a	n/a	n/a	n/a	n/a	n/a
Gas Dehydrator	9	7	8	n/a	n/a	n/a	n/a	n/a	n/a	n/a	n/a	n/a	6	5	5	67	100	100
Gas Processing Plant	77	76	74	74	72	71	88	93	92	77	81	92	n/a	n/a	n/a	n/a	n/a	n/a
Gas Sales Meter	134	126	119	n/a	n/a	n/a	n/a	n/a	n/a	n/a	n/a	n/a	125	110	110	78	91	91
Injection Station	6	6	6	4	5	5	75	40	40	100	80	80	n/a	n/a	n/a	n/a	n/a	n/a
NGL Fractionation Facility	1	1	1	n/a	n/a	n/a	n/a	n/a	n/a	n/a	n/a	n/a	1	1	1	100	100	100
Oil Sales Meter	53	53	51	n/a	n/a	n/a	n/a	n/a	n/a	n/a	n/a	n/a	47	50	49	100	100	100
Pipeline Gathering	2	1	1	n/a	n/a	n/a	n/a	n/a	n/a	n/a	n/a	n/a	1	1	1	100	100	100
Processing Battery	42	41	37	37	33	31	81	85	94	70	88	100	n/a	n/a	n/a	n/a	n/a	n/a
Satellite Battery	105	137	148	12	10	12	42	50	58	67	90	83	n/a	n/a	n/a	n/a	n/a	n/a
Tank Terminal	9	9	9	7	7	8	86	86	88	100	100	100	n/a	n/a	n/a	n/a	n/a	n/a
Water Hub	10	15	12	n/a	n/a	n/a	n/a	n/a	n/a	n/a	n/a	n/a	10	13	11	70	77	91
Total Facilities	**814**	**807**	**787**	**446**	**400**	**390**	**85**	**95**	**96**	**65**	**80**	**85**	**190**	**180**	**177**	**83**	**93**	**94**
**Total Wells**	**9947**	**10258**	**10649**	**6212**	**6714**	**7194**	**78**	**95**	**95**	**78**	**95**	**95**	**2950**	**2918**	**2985**	**57**	**81**	**86**
**Total**	**10761**	**11065**	**11436**	**6658**	**7114**	**7584**	**78**	**95**	**95**	**77**	**94**	**95**	**3140**	**3098**	**3162**	**59**	**82**	**87**

* Facilities active (pressurized) more than 30 days per year and wells active more than 90 days per year are subject to LDAR regulations at prorated survey frequency as per Table S3.

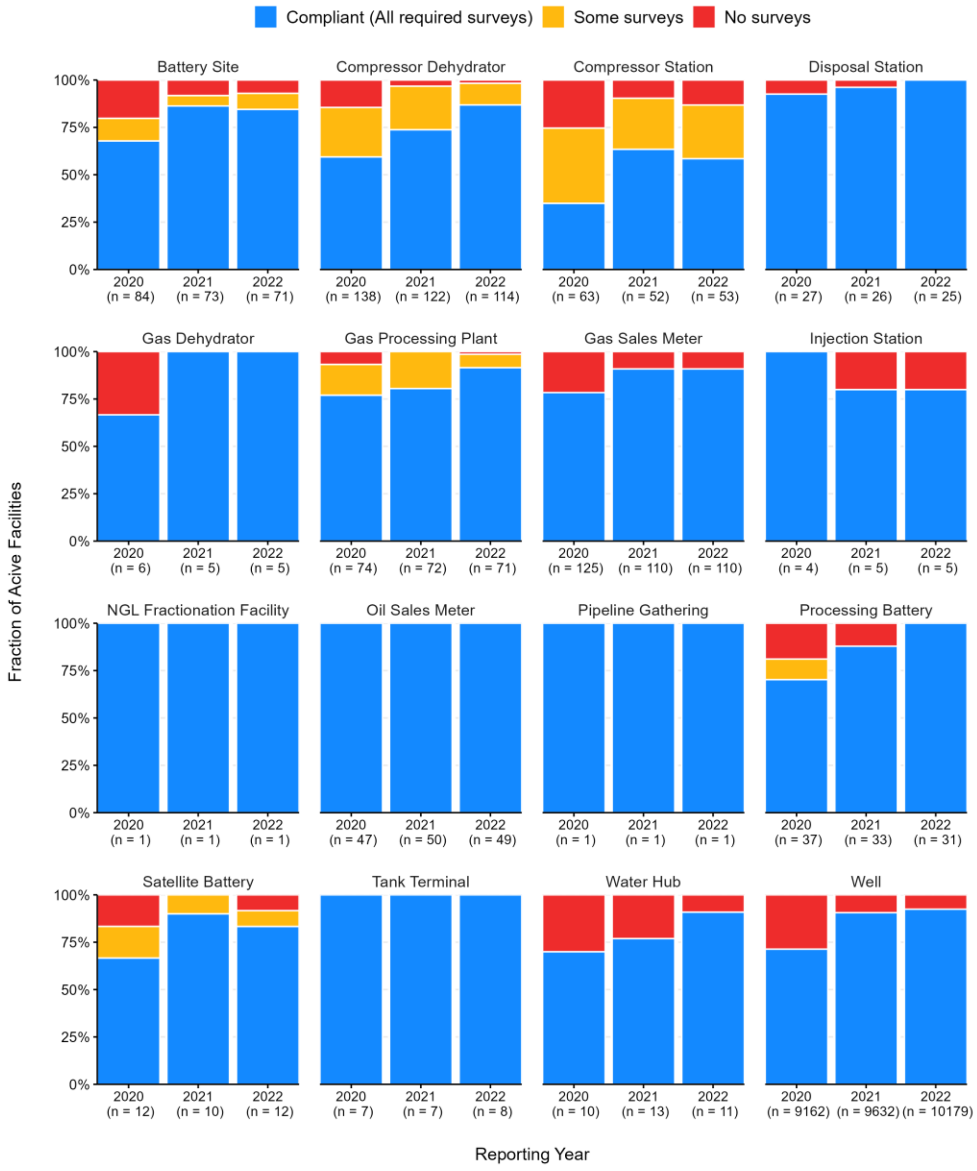

Figure S2: Summary compliance statistics for each facility type split by reporting year.

S1.5 Compliance Based on Facility or Well Sites (Pads)

In practice, co-located facilities and wells are often treated as a single "site" where all infrastructure is situated on one "pad" (i.e., one single cleared and graded area), belonging to a single operator. During LDAR surveys, it is expected that leaks are reported to an individual facility, defined by a KERMIT ID, or well, defined by a WA number. However, this approach can be impractical because it's not always clear which infrastructure corresponds to each facility or well. An alternative would be to treat each site (pad)

as a single entity, requiring a single type (comprehensive or screening) and number of LDAR surveys per year.

For the set of 5,835 polygons defined in S1.3, we assigned a required number and type of LDAR survey during each year based on the entities present on each site. The enclosed KERMIT facility or well requiring the highest number of annual surveys was used to determine the number of required surveys for the entire site, where comprehensive surveys take precedence over screening surveys. For example, for a site containing a Compressor Station and a Gas Sales Meter, the Compressor Station would dictate that 3 comprehensive surveys per year were required. A site was defined as a facility if it contained one or more facilities identified with a KERMIT ID along with any number of wells. Well sites were defined as polygons containing only wells and no facility infrastructure. Compliance statistics were then calculated for the polygon (pad) in the same manner as in S1.3. These final compliance statistics by site are presented in Table S9 and Figure S3 and are the underlying data presented as Figure 1 of the main text.

Table S9: Compliance determined for active facility and well *sites*, defined by a polygon encompassing all infrastructure on a single pad, split by the type of site/pad (facility or well) and type of survey required (comprehensive or screening). Compliance was calculated on *a per-pad basis*.

Year	Required Survey Type	*Facility Sites*						*Well Sites*	
		Number Active during >30 days of year*	Number Requiring Surveys at:			Fraction with at least one Survey	Fully Compliant Fraction (with ALL required surveys)	Number Active >90 days per year, requiring 1×/yr surveys*	Fully Compliant Fraction (fraction surveyed)
			3×/yr	2×/yr	1×/yr				
**2020**	Comprehensive	411	270	10	131	83	64	2210	68
	Screening	16	-	-	16	44	44	2260	49
**2021**	Comprehensive	391	249	3	139	95	80	2222	92
	Screening	14	-	-	14	57	57	2206	80
**2022**	Comprehensive	378	243	-	135	96	86	2291	91
	Screening	18	-	-	18	67	67	2243	83

* Facilities active (pressurized) more than 30 days per year and wells active more than 90 days per year are subject to LDAR regulations at prorated survey frequency as per Table S3.

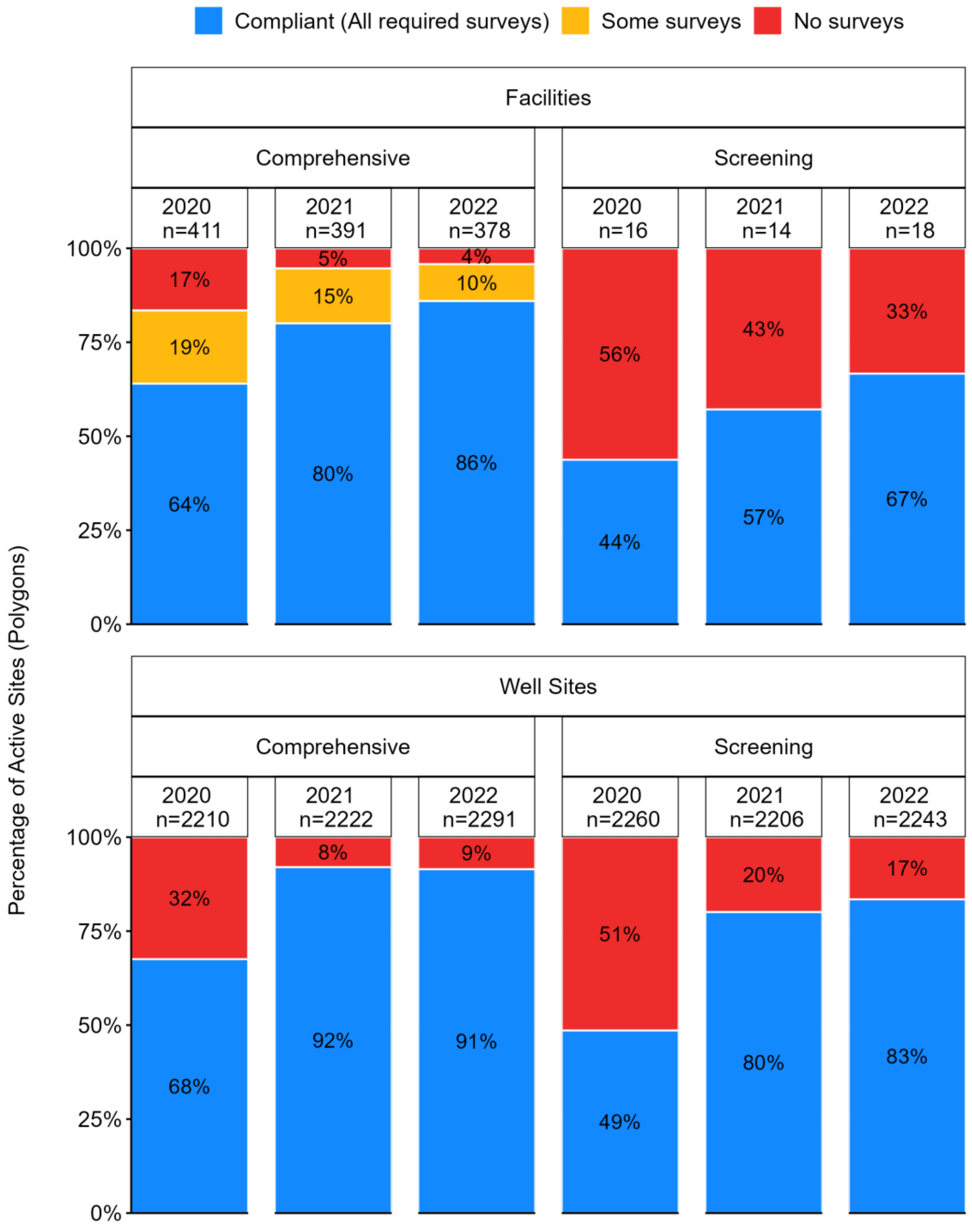

Figure S3: Compliance statistics for active facility and well *sites*, defined by a polygon encompassing all infrastructure on a single pad, split by the type of site/pad (facility or well) and type of survey required (comprehensive or screening). Compliance was calculated on *a per-pad basis*. Readers are cautioned on the small number (n=<18) of facilities only requiring screening surveys (upper right panel) such that the percentages for this category are arguably less meaningful.

S2 Calculating the Change in Emissions Over Time Considering the Level of Compliance

The rate of compliance directly impacts the extent of emission reductions achieved by any LDAR program. Calculating a year-over-year change in total emissions found during LDAR surveys could be misleading, as it only includes sites that were actually surveyed. In theory, significant emission reductions could result from high compliance in the first year of surveys, followed by poor compliance in subsequent years, leading to a decrease in the number and magnitude of detected leaks. Similarly, overall fugitive emissions might be underestimated if only a portion of sites are fully compliant with regulations. To address this, emissions for sites with "missing" surveys (i.e. those not conducted or submitted due to non-compliance) were estimated during the time period of any missing surveys. This enabled analysis of emission trends that explicitly accounted for the effects of non-compliance.

First, for all operating facility and well sites (as determined above in S1.1) a list of all required surveys was compiled. Surveys were labeled numerically according to the survey year, where year 1 represents the first year of required reporting for that facility or well site. For example, for a facility that was not operating until 2021, year 1 and year 2 would correspond to 2021 and 2022, respectively. Consecutive surveys conducted within the same reporting year were labeled alphabetically, so that regardless of the survey date, the first survey was labelled as A, the second as B, and the third as C. For instance, a Compressor Station operating throughout 2020 to 2022, which requires three surveys each year, would require surveys labeled 1A, 1B, 1C, 2A, 2B, 2C, 3A, 3B, and 3C. By contrast, well sites that require only one survey per year would require surveys labeled 1A, 2A, and 3A.

Total emissions for missing surveys were imputed as follows:

- Whenever possible, missing survey emissions were estimated based on the total emissions detected during the next survey that took place at that facility or well. This assumes that, in the absence of repairs, any emissions found during a subsequent survey would have been present during the missed survey.
- If there were no subsequent surveys, the emissions for the missing survey were calculated as the average total emissions for that particular survey number. For example, if a facility was missing survey 3C, the total emissions would be estimated as the average of all 3C surveys.
- In the case where a facility or well lacked two or more consecutive surveys with no subsequent survey, (e.g. a facility missing surveys 3B and 3C), then both surveys were estimated as the average of the first missing survey as no repairs would have been conducted and emissions would likely have remained unchanged from one survey to the next.

S3 Additional Figures of Analyzed Reported LDAR Data

When plotting emission rates, reported volumetric leak rate data in m^3/h were assumed to be at oil industry standard conditions of 15°C at 101.325 kPa and converted to units of kg/h assuming that the measured gases were methane (in the absence of specific leak composition data).

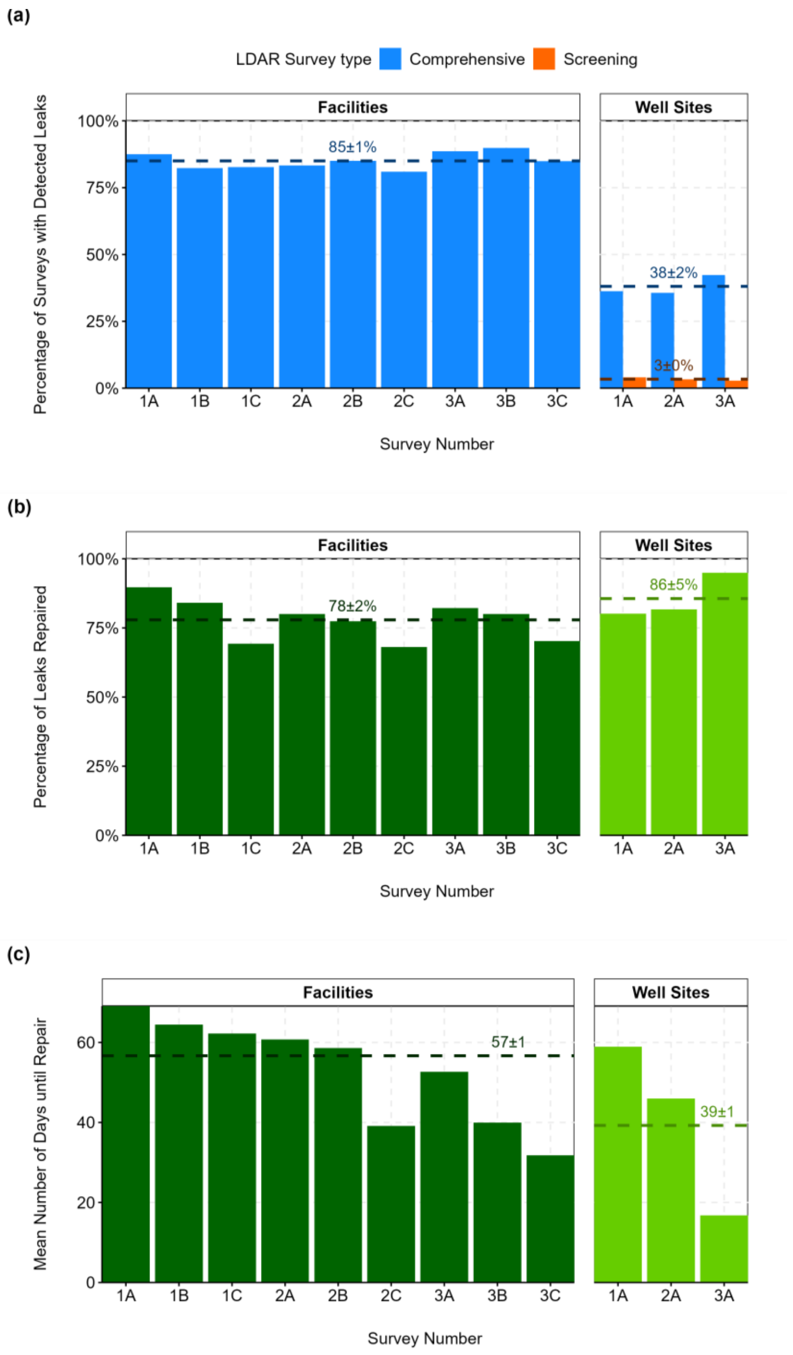

Figure S4: Detected leak and repair statistics for all facility and well sites surveyed between 2020-2022 as a function of survey number showing (a) the percentage of surveys during where at least one leak was detected, (b) the percentage of leaks that were repaired based on reported repair dates, and (c) the mean number of days taken to repair a detected leak. The dashed lines represent the mean values calculated across all surveys.

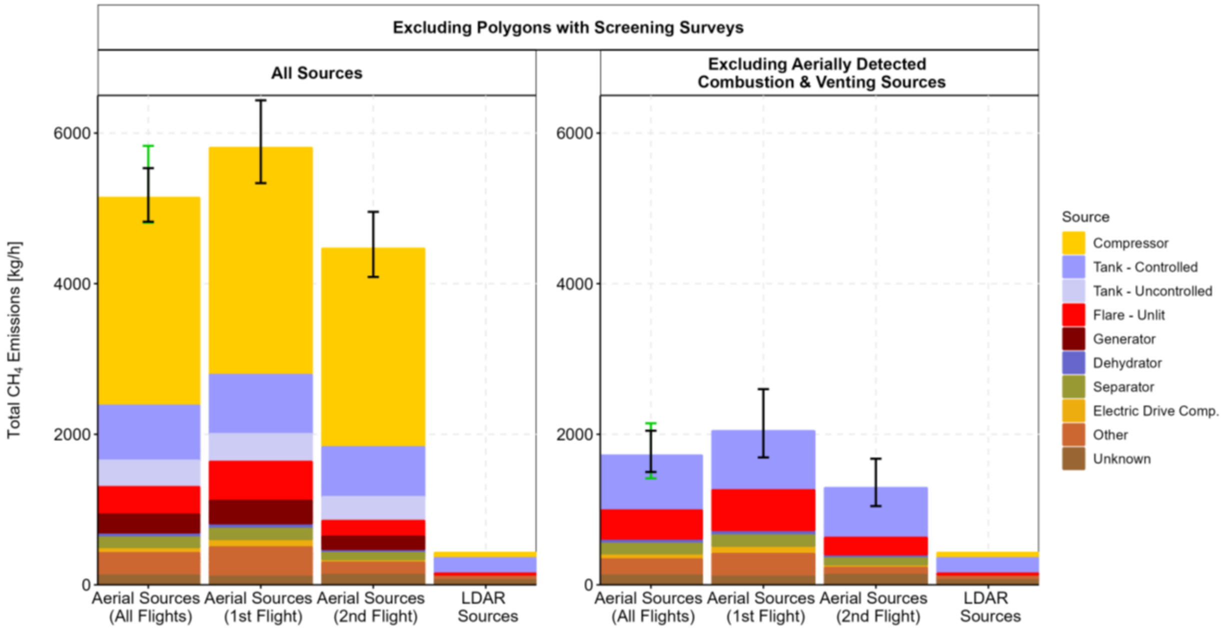

Figure S5: Comparison of total aerially measured emissions determined using all flight days, only data acquired during the first flight over each source, or only data from the second flight over each source (acquired on a different day 1-10 days after the first flight) with LDAR measured emissions at the same set of 326 sites (pads) in 2021. Note that measurements during each flight day may include up to five passes over the same source. Error bars (black) represent the 95% confidence interval calculated using a Monte Carlo analysis, with the estimated added uncertainty from source intermittency shown in green following the method detailed in (Johnson et al., 2023).

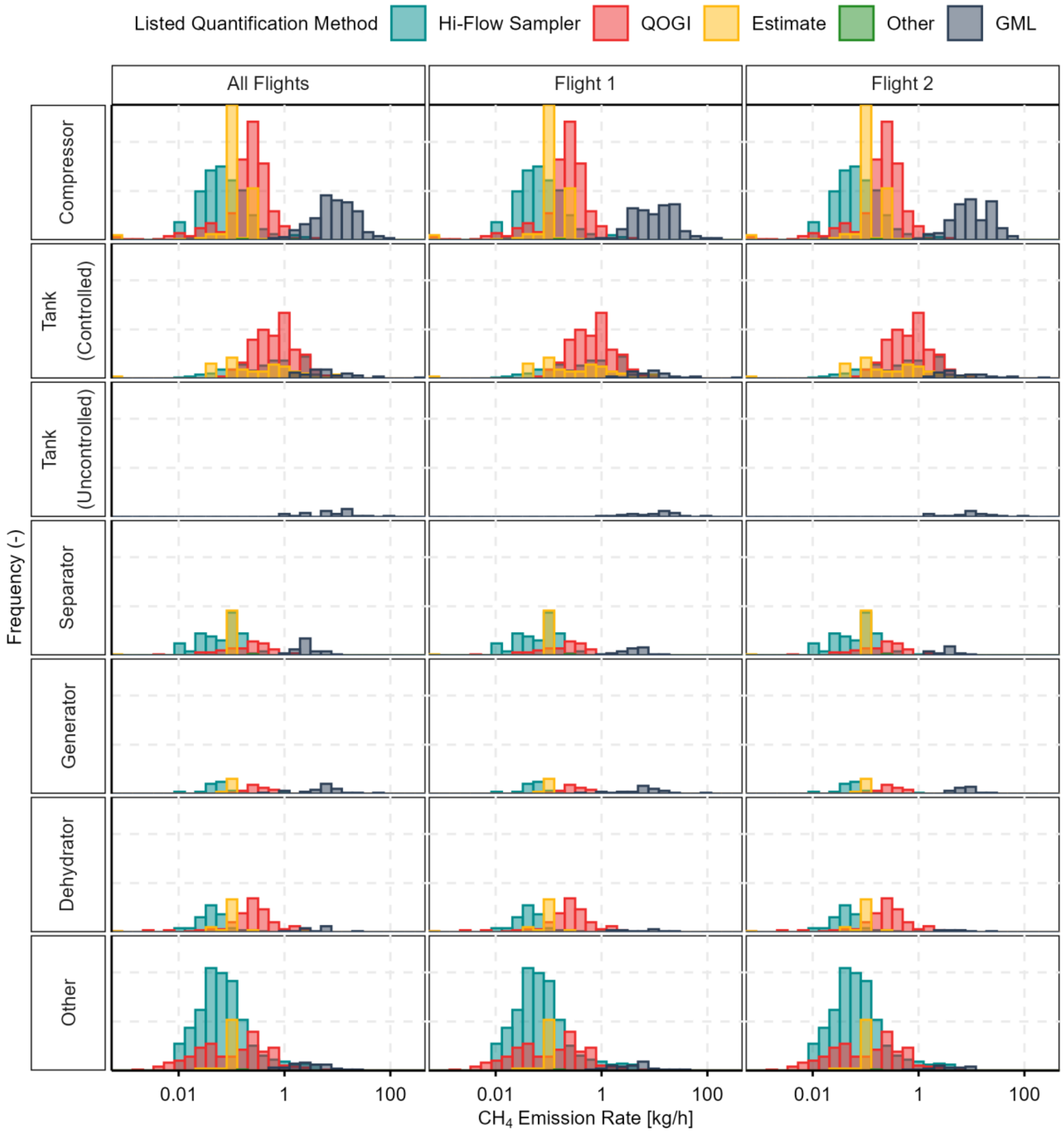

Figure S6: Distributions of methane emission rates detected during the LDAR and aerial surveys at the same subset of 326 sites (pads) with comprehensive LDAR surveys in 2021 colored by the reported quantification method. Leaks detected during LDAR surveys were quantified using Hi-Flow Sampler, QOGI, engineering calculations, emission factors, or other approaches. Aerial surveys used Bridger Photonics' Gas Mapping LiDAR. Rows distinguish distributions for different equipment types (Compressors, Controlled Tanks, Uncontrolled Tanks, Separators, Generators, Dehydrators, and Other) and columns include aerial data from all flights, from flight 1 only, or from flight 2 only. Flight 1 represents measurements during the first flight day over a source, while flight 2 corresponds to measurements during the second flight day which occurs 1–10 days later.

Table S10: Comparison of source counts and total methane emissions in 2021 at the same subset of 326 sites (pads) as seen via aerial gas mapping LiDAR (GML) and via comprehensive LDAR surveys, for each major equipment source category. LDAR data are the average detected amounts and counts over all available surveys for each site during 2021 (See also Section 3.3 of the main manuscript).

Source	Total CH_4 Emissions [kg/h]			Total Source Count	
	Aerial	LDAR	Factor (Aerial/LDAR)	Aerial	LDAR
**Aerial Sources in Figure 3b**					
Tank - Controlled	729.7	204.1	3.6	42	217
Separator	108.4	16.4	6.6	34	115
Dehydrator	77.6	18.7	4.2	14	83
Other	154.8	61.5	2.5	37	306
Unknown	118.5	10.4	11.4	19	11
Flare - Unlit	364.8	3.1	116.0	14	2
Meter	16.2	12.5	1.3	6	56
Wellhead	7.5	5.2	1.4	3	51
Flare System	7.4	6.9	1.1	1	13
Electric Drive Comp.	89.8	-	∞	7	-
Fracturing Equipment	22.6	0.1	215.9	1	1
Truck Loading	16.1	-	∞	1	-
Subtotal	*1713.4*	*338.9*	*5.1*	*179*	*855*
**Additional Aerial Sources in Figure 3a**					
Compressor	2761.0	71.8	38	219	357
Tank - Uncontrolled	351.0	-	-	28	-
Generator	227.6	4.2	54	28	29
Heater	44.9	5.8	8	8	52
Vent Stack	30.6	-	-	2	-
Flare - Lit	7.1	-	-	2	-
Subtotal	*3422.2*	*81.8*	*41.8*	*287*	*438*
**Total (All Sources)**	**5135.6**	**420.7**	**12.2**	**466**	**1294**
**Total (Only Aerial Sources from Figure 3b vs. ALL LDAR sources)**	**1713.3**	**420.7**	**4.1**	**179**	**1294**

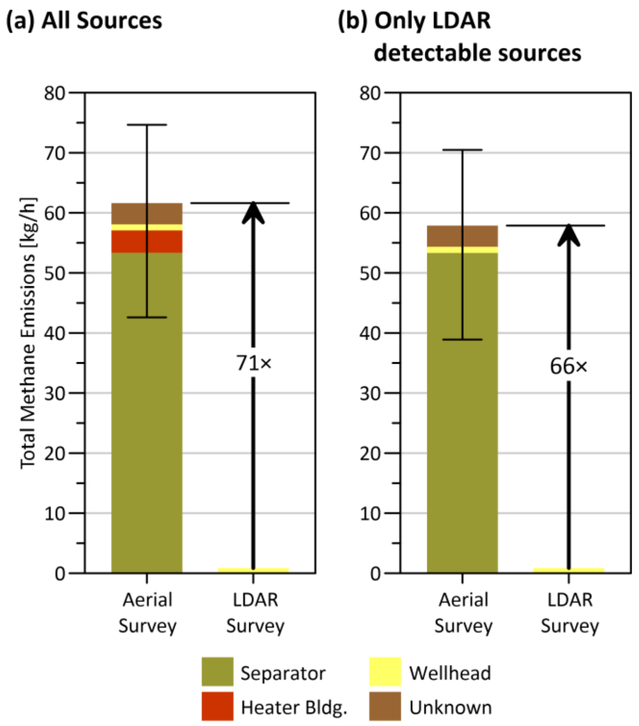

Figure S7: Contrast in aerial measured methane emissions and reported emissions in LDAR reports from screening surveys at an identical set of 59 sites in 2021.

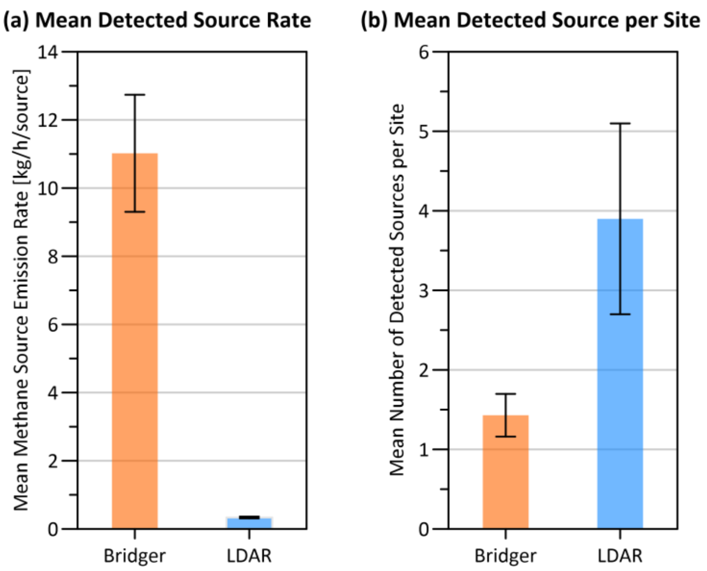

Figure S8: Leak statistics from aerial and LDAR surveys (first surveys only) for the same subset of 326 sites (pads) with comprehensive LDAR surveys showing (a) the mean source emission rate and (b) the mean number of leaks detected per site (polygon). Error bars in the mean aerially-measured leak rate (a) were calculated using a Monte Carlo simulation that accounts for quantification error of the GML technology, whereas the LDAR data error bar is simply the 95% confidence interval in the mean assuming no error in quantified emission rates. For the source count data in (b), the error bars simply represent a 95% confidence interval in the mean.

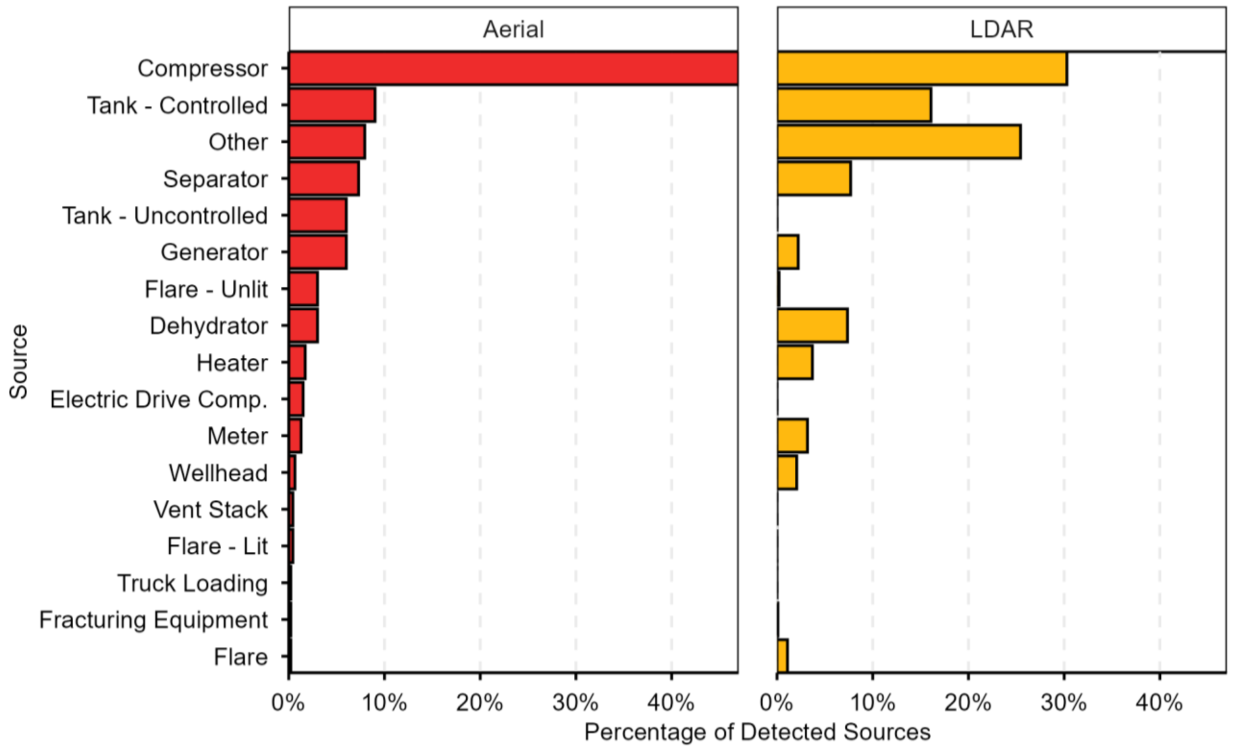

Figure S9: Frequency of individual emission sources detected during aerial and comprehensive LDAR surveys at the same subset of 326 sites (pads).

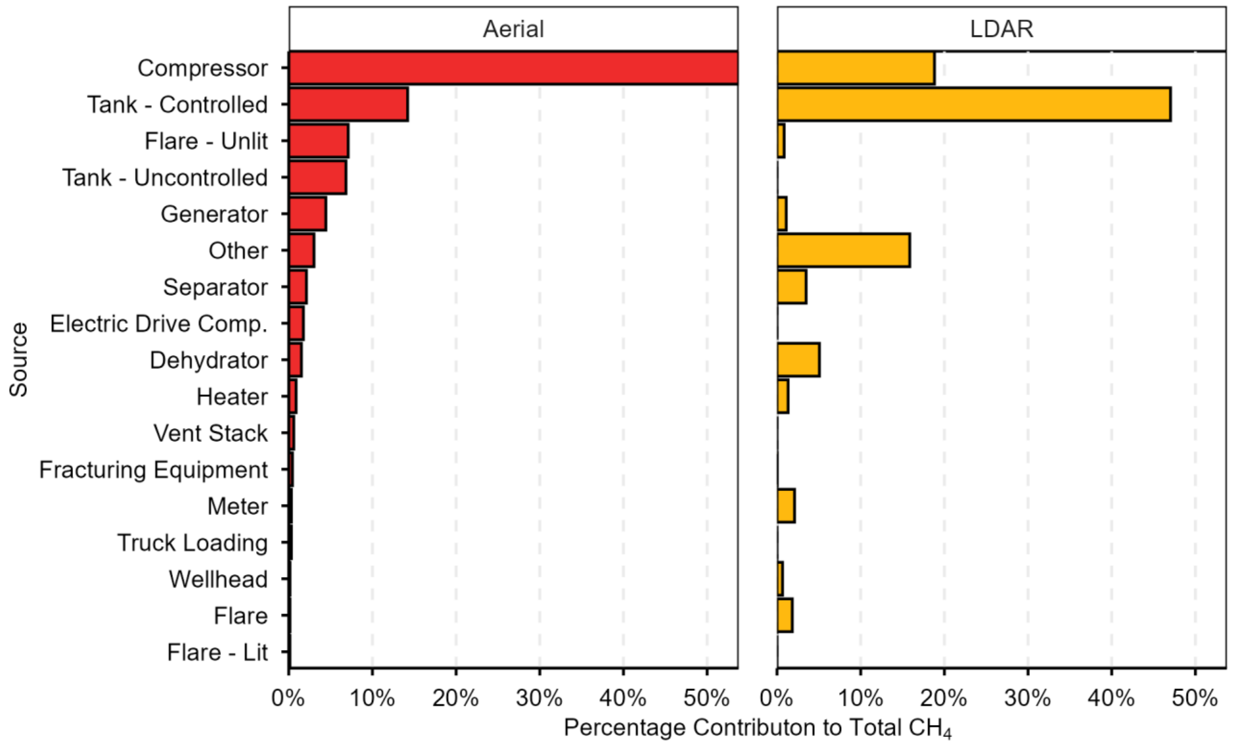

Figure S10: Percentage contribution of individual sources to total methane emissions measured at the same subset of 326 sites (pads) during aerial and comprehensive LDAR surveys.

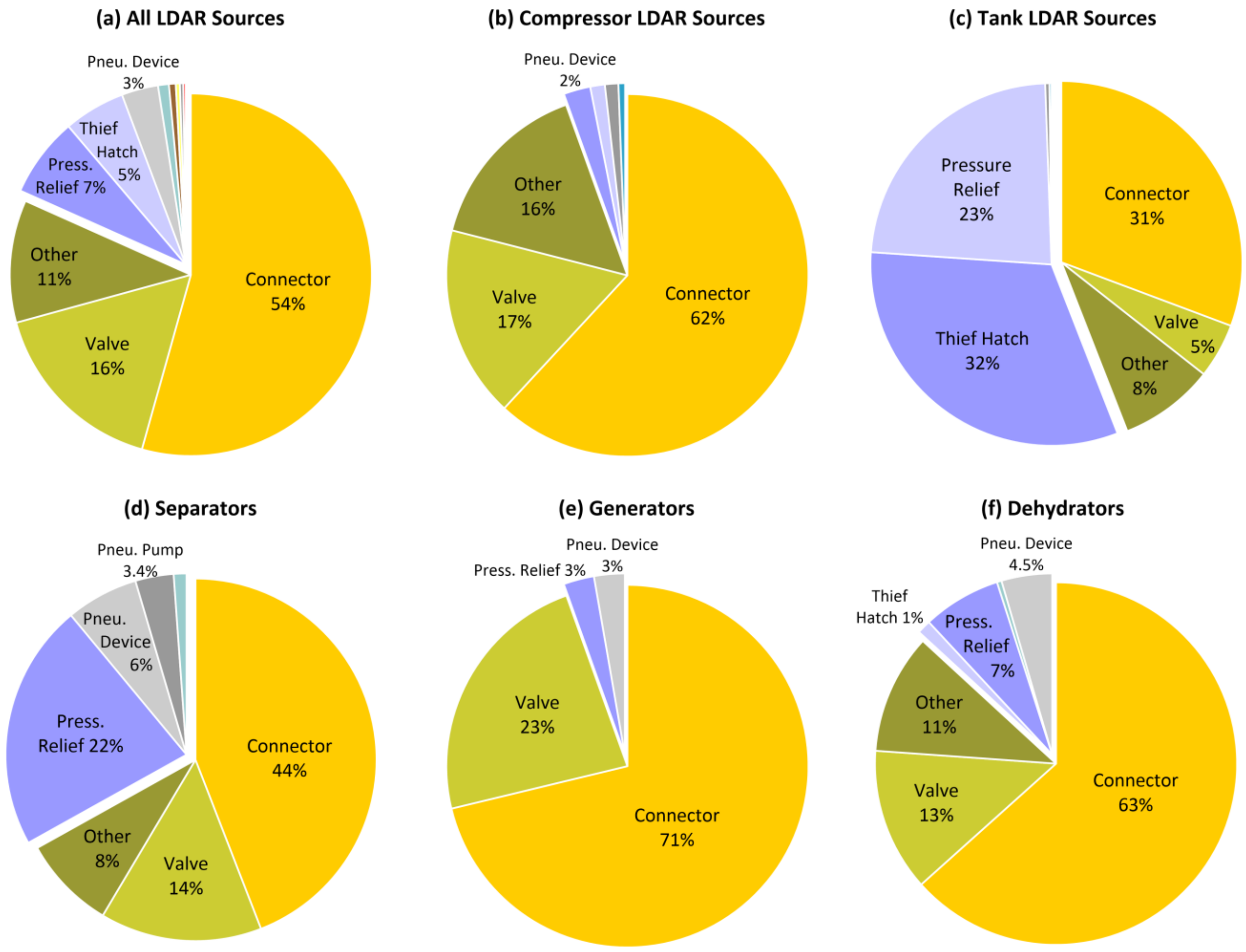

Figure S11: Breakdown of emitting sources detected in comprehensive LDAR surveys at a subset of 326 sites (pads): (a) all LDAR sources, (b) LDAR sources associated with compressors, (c) LDAR sources associated with tanks, (d) LDAR sources associated with separators, (e) LDAR sources associated with generators and (f) LDAR sources associated with dehydrators. Sources classed as "other" include leaking regulators, meters, and sources not specified in the available LDAR reports.

S4 References

BCER, (2019). Fugitive Emissions Management Guideline. INDB 2019-18, British Columbia Energy Regulator (BCER). Available at: https://www.bc-er.ca/news/new-fugitive-emissions-management-guideline/

BCER, (2021). Facility Inventory [BCOGC-41090]. https://www.bc-er.ca/data-reports/data-centre/?category=41240 (accessed October 16, 2023)

BCER, (2023a). 2021 Equivalency Report. British Columbia Energy Regulator (BCER). Available at: https://www.bc-er.ca/files/reports/Methane-Equivalency-Reports/Equivalency-Report_FINAL_March2023.pdf

BCER, (2023b). BC Total Production [BCOGC-41143]. https://reports.bc-er.ca/ogc/f?p=200:8:15361179614398::NO::: (accessed January 4, 2024)

BCER, (2023c). Well Index [BCOGC-2555]. https://www.bc-er.ca/data-reports/data-centre/?category=2772 (accessed November 22, 2023)

BCER, (2023d). Regional Fields [BCOGC-44808]. https://data-bc-er.opendata.arcgis.com/datasets/2e34e8d9065a46929a9dfdbf97ad3838_1/about

BCOGC, (2021). *Oil and Gas Activities Act Drilling and Production Regulation*. British Columbia, British Columbia Oil and Gas Commission (BCOGC).

Johnson, M.R., Conrad, B.M., and Tyner, D.R., (2023). Creating measurement-based oil and gas sector methane inventories using source-resolved aerial surveys. *Commun. Earth Environ.*. 4:139.

Petrinex, (2022). Petrinex Activity Codes (British Columbia). https://www.petrinex.gov.ab.ca/bbreportsBC/PRAActivityCodes.htm (accessed October 18, 2022)

Petrinex, (2023). Petrinex Facility and Sub-types Codes Report (Alberta). https://www.petrinex.gov.ab.ca/bbreports/PRAFacilityCodes.htm (accessed May 29, 2023)
